# Contact modulation in NbX_2_–HfY_2_ (X = Se, Te; Y = S, Se) van der Waals heterostructures: Schottky-to-Ohmic transition

**DOI:** 10.1039/d6na00658b

**Published:** 2026-09-10

**Authors:** Johar Ali, Sahibzada I. H. Bacha, M. Bilal, B. Amin

**Affiliations:** a Department of Physics, Abbottabad University of Science & Technology Abbottabad 22010 Pakistan binukhn@gmail.com +92-333-943-665; b Department of Physics, Government Post-Graduate College Mardan 23200 Pakistan

## Abstract

Extensive efforts have been directed toward exploring novel metal–semiconductor (MS) interfaces and engineering their contact properties to minimize contact resistance. The electronic structure and interfacial properties of van der Waals heterostructures (vdWHs) composed of metallic NbX_2_ (X = Se, Te) and semiconducting HfY_2_ (Y = S, Se) monolayers are systematically investigated *via* first-principles calculations. Binding energy analysis, phonon dispersion spectra, and Born mechanical stability criteria are used to verify the structural integrity, thermodynamic stability, and experimental feasibility of these materials. *Ab Initio* Molecular Dynamics (AIMD) simulations also confirm the thermal stability of the NbX_2_–HfY_2_ vdWHs at 300 K, with no significant structural reconstruction or bond breakage observed over a 4 ps timescale. These investigations reveal that the vdWHs preserve the intrinsic electronic characteristics of their constituent layers due to weak vdW coupling, while benefiting from the high metallic conductivity of the NbX_2_ layer. At equilibrium, the interfaces predominantly form n-type Schottky contact, where a significant interfacial dipole potential modulates the band alignment. These heterostructures exhibit highly tunable behavior; specifically, mechanical deformation triggers a transition from Schottky to Ohmic contacts, facilitating high-efficiency carrier injection. This transition is driven by enhanced orbital hybridization between Nb-d and Hf-d states near the Fermi level. While HfS_2_-based systems retain a robust n-type character, the HfSe_2_ counterparts display higher sensitivity to band shifting under strain. These findings provide a comprehensive theoretical framework for utilizing NbX_2_–HfY_2_ (X= Se, Te; Y = S, Se) MS contacts in next-generation, low-resistance 2D field-effect transistors and flexible nanoelectronics.

## Abstract 2

Extensive efforts have been directed toward exploring novel metal–semiconductor (MS) interfaces and engineering their contact properties to minimize contact resistance.

## Graphical abstract

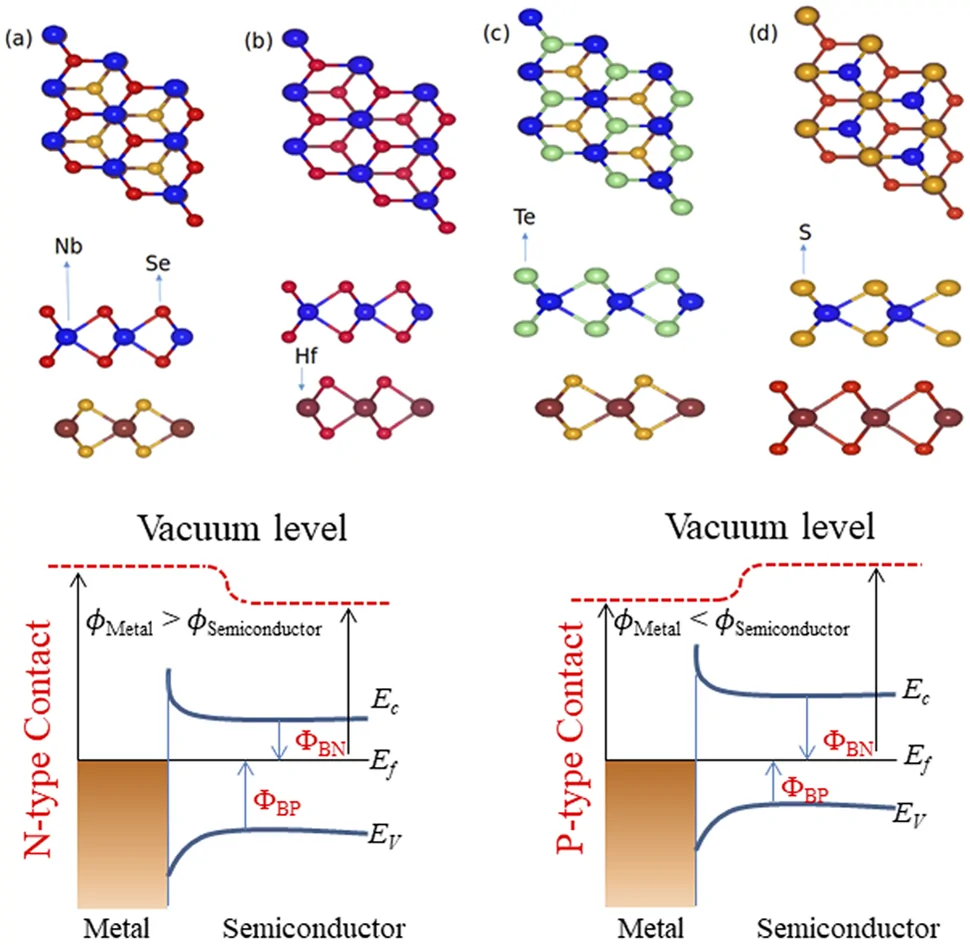

## 1. Introduction

Atomically thin structural design, elevated carrier mobility, and extraordinary mechanical flexibility make two-dimensional (2D) transition metal dichalcogenides (TMDs) favorable candidates in device applications.^1,2^ However, the performance of these devices are severely limited by the contact resistance at metal–semiconductor (MS) interfaces,^3,4^ which originates from Schottky barriers^5^ and strong Fermi-level pinning (FLP).^6,7^ Additionally, atomic-scale defects generated during fabrication of practical devices also complicate the regulation of the Schottky barrier height (SBH).^8,9^

These challenges are resolved by using approaches, like MS van der Waals (vdW) contact engineering,^10^ doping^11^ and using semimetallic electrodes.^12^ van der Waals contacts in the form of heterostructures (vdWHs)^13,14^ composed of metallic and semiconducting 2D layers have emerged as a promising platform for achieving high-quality, low-resistance interfaces without the complications of chemical bonding or lattice mismatch.^15^ MS vdW contacts precisely control the transport of carriers and hence find potential applications in nanoelectronic devices.^16–18^ Therefore, in the family of 2D TMDs, metallic NbX_2_ (X = Se, Se) and semiconducting HfY_2_ (Y = S, Se) offer complementary electronic properties that make them ideal candidates for vdW MS contact engineering.^19,20^

Beyond material selection, strain engineering is also found to be an effective experimentally viable strategy for modulating the electronic structure and SBH properties at 2D interfaces.^21–23^ A tensile strain of about 0.7% is used to reduce SBH^24^ at MoS_2_/Au interfaces. Similarly, contact resistance in other 2D systems^25,26^ has also been tuned by strain engineering. The mechanisms beyond the strain-induced modulation of SBH in vdWHs particularly those involving metallic NbX_2_ and semiconducting HfY_2_ remain largely unexplored. In contrast to conventional TMD/metal interfaces,^27^ where strong chemical interactions dominate, the weak vdW coupling in NbX_2_–HfY_2_ heterostructure (vdWHs) introduces distinct interfacial physics, including different charge transfer mechanisms, orbital hybridization pathways, and strain responses. Furthermore, while most existing studies focus on uniaxial strain, the effect of biaxial strain,^28,29^ which is more commonly encountered in flexible device applications, on SBH modulation in these vdWHs has not been systematically investigated.

In this work, NbX_2_–HfY_2_ MS vdWHs are modelled, while their interfacial properties, like the electronic band structure, charge dynamics and type and height of the barrier, are investigated *via* first principles calculation. Strain engineering is used to tune the bond lengths, orbital overlapping and density of states at the interface that affect the carrier transport. The modelled NbX_2_–HfY_2_ MS vdWHs in these findings have an adjustable SBH, and their contact type is tuned from p-type to n-type and from Schottky to Ohmic under strain, and hence they are found to be potential candidates for nanoscale electronic devices. These findings provide a comprehensive theoretical framework for utilizing NbX_2_-based contacts in next-generation, low-resistance 2D electronics.

## 2. Computational details

Density functional theory (DFT) calculations in the PWSCF code^30^ with the Perdew–Burke–Ernzerhof (PBE) exchange-correlation functional^31^ along with the Grimme's DFT-D2 dispersion corrections^32^ are carried out. Using a plane-wave cutoff energy of 700 eV, a Monkhorst–Pack *K*-point grid of 8 × 8 × 1 (16 × 16 × 1) is used for geometry optimization (self-consistent field calculations). The convergence thresholds are set to 10^−3^ eV Å^−1^ for forces and to 10^−4^ eV for energy calculations. A vacuum space of 25 Å was introduced in the *z*-direction to eliminate interactions between periodic images. Phonon spectra confirm the dynamic stability,^33^ while the thermal stability was further examined *via ab initio* molecular dynamics (AIMD) simulations at 300 K over a duration of 4 ps with a 1 fs time step^34,35^ and the mechanical stability was examined through the energy strain approach.^36^

Binding energies for all stacking configurations of NbX_2_–HfY_2_ (X = Se, Te; Y = S, Se) are calculated *via*^37^*E*_b_ = *E*_vdWH_ − *E*_semiconducting-layer_ − *E*_metallic-layer_, where *E*_vdWH_, *E*_semiconducting-layer_ and *E*_metallic-layer_ are the energies of the vdWHs and isolated semiconducting and metallic layers, respectively. The charge density differences are calculated *via*^38^ Δ*ρ* = *ρ*_(vdWHs)_ − *ρ*_(semiconducting-layer)_) − *ρ*_(metallic-layer)_, where *ρ*_(vdWHs)_, *ρ*_(semiconducting-layer)_) and *ρ*_(metallic−layer)_ are the charge densities of the vdWHs and their respective constituent layers. Effective mass of the carriers is computed *via*
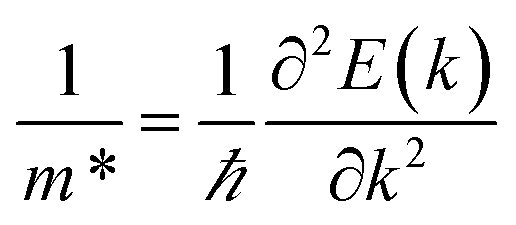
 by parabolic fitting of the CBM and VBM,^39,40^ where *ℏ* is the Planck constant, *E*(*K*) is the dispersion relation, and *K* is the wave vector near the CBM and VBM. SBH was assessed using both the Schottky–Mott (SM) rule *Φ*_B_p__ = *I* − *ϕ*_metal_ and *Φ*_B_n__ = *ϕ*_metal_ − *χ* (*I* is the ionization energy of the isolated semiconductor monolayer, *χ* is its electron affinity, and *ϕ*_metal_ is the work function of the pristine metal monolayer)^41,42^ and first-principles calculations *Φ*_B_p__ = *E*_F_ − *E*_VBM_ and *Φ*_B_n__ = *E*_CBM_ − *E*_F_ (the energy level of the valence band maximum is *E*_VBM_ and conduction band minimum is *E*_CBM_ of the semiconductor within the heterostructure, respectively). The degree of the FLP, induced by the interface dipole (ID), was analyzed *via* a slope parameter *S*; *S* = |∂*Φ*_Bp_/∂*ϕ*_m_|; *ϕ*_m_ is the metal work function.^43^ Tunneling barrier probability^44^
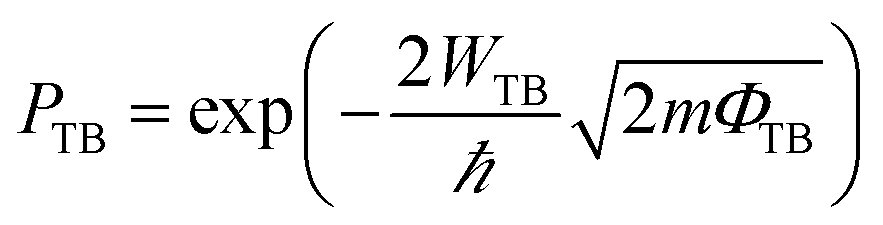
 in terms of the comprehensive factor is *C* (*C* = (*W*_TB_)^2^*Φ*_TB_. Here, *W*_TB_ is the physical tunneling barrier width (extracted as the full-width at half-maximum of the electrostatic potential barrier at the interface), Φ_TB_ is the height of the interfacial tunneling potential barrier relative to the Fermi level, and m is the free electron rest mass.

## 3. Results and discussion

In agreement with ref. 45, the calculated electronic band structure and density of states of NbX_2_ (X = Se, Te) and HfY_2_ (Y = S, Se) monolayers validate the reliability of the present approach for the subsequent heterostructure analysis. The calculated lattice mismatch of the corresponding monolayers is 1.41% for NbSe_2_–HfS_2_, 4.90% for NbSe_2_–HfSe_2_, 3.27% for NbTe_2_–HfS_2_, and 0.27% for NbTe_2_–HfSe_2_,^46–48^ confirming the experimental feasibility of constructing these vdWHs with minimal interfacial strain. Notably, NbTe_2_–HfSe_2_ exhibits nearly perfect lattice matching (0.27%), making it an ideal candidate for high-quality vdW interface fabrication. Therefore, using a 1 × 1 unit cell of each NbX_2_ and HfY_2_ monolayers, NbX_2_–HfY_2_ MS vdWHs are modeled in four distinct stacking configurations, as illustrated in Fig. 1. The interlayer distances (*d* ∼2.55–4.95 Å) and binding energies (*E*_b_ ∼−0.0135–−0.0513 eV), in Table 1, confirm the thermodynamic stability of all four configurations. The larger “*d*” than typical covalent bond lengths implies that the intrinsic electronic properties of the isolated NbX_2_ (X = Se, Te) and HfY_2_ (Y = S, Se) monolayers remain intact within the vdWHs, an essential feature for the development of next-generation nanoelectronic devices.^49^ The highest (shortest) *E*_b_(*d*) shows that the preferred stacking is (*b*) for all NbX_2_–HfY_2_ MS vdWHs. The optimized lattice constant and bond length of the most favorable stacking configuration are listed in Table 2.

**Fig. 1:**
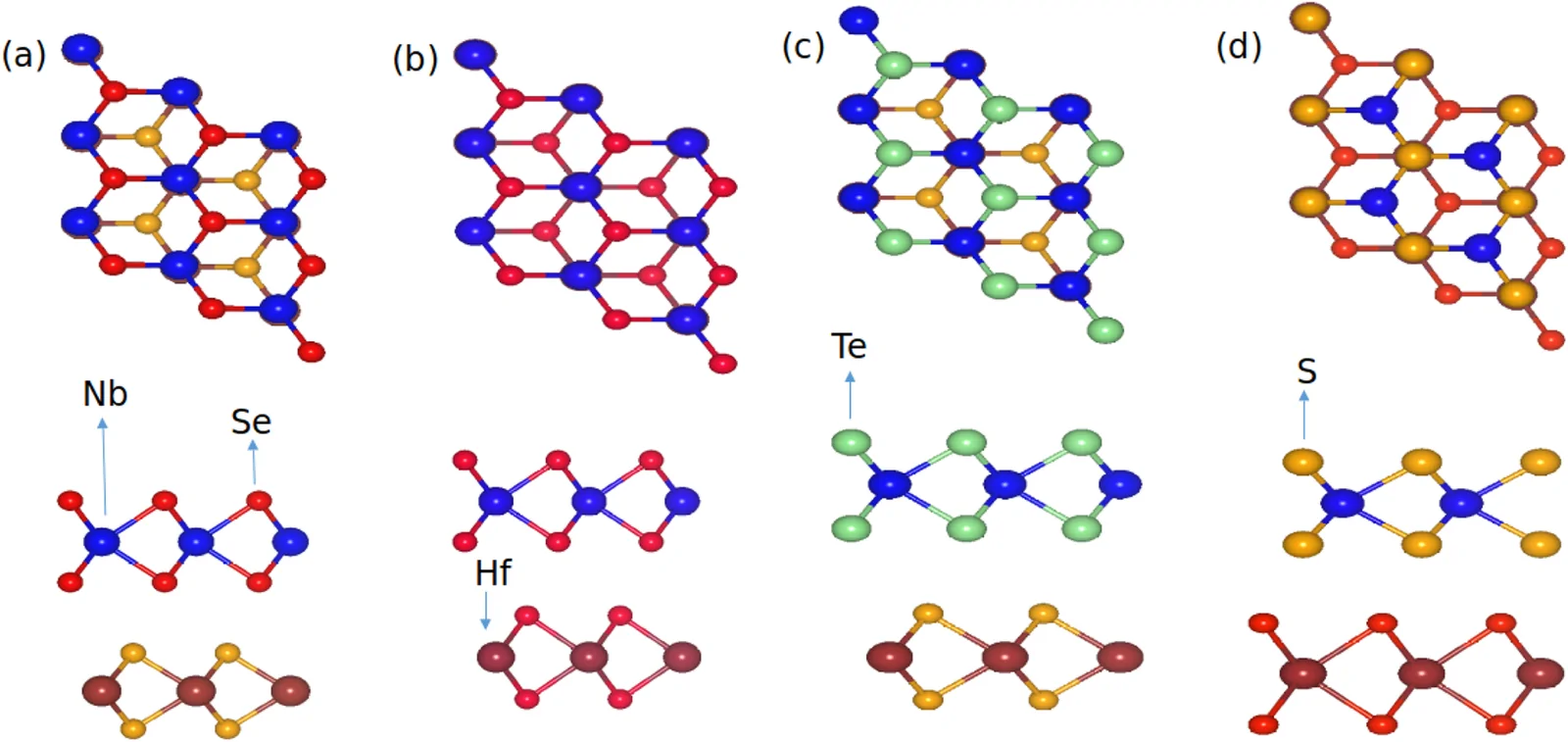
Stacking configuration for NbX_2_-HfY_2_ (X = Se, Te; Y = S, Se) MS vdWHs. (a) Se atom of the NbSe_2_ layer is positioned directly above the hexagonal center of HfS_2_ layer, while the Nb atom is vertically aligned with the S atom (b) Se atom of NbSe_2_ is aligned directly over the Se atom of the HfSe_2_ layer (c) Te atom sits directly above the S atom of the HfS_2_ layer, while the Nb atom is aligned over the Hf atom (d) Te atom of NbTe_2_ is aligned directly over the Se atom of HfSe_2_, while the Nb atom is positioned over the hexagonal centre.

**Table 1:** Binding energies (*E*_b_ in eV) and interlayer distance (*d* in Å) of NbX_2_–HfY_2_ (X = Se, Te; Y = S, Se) vdWHs in stacking ((a)–(d))

NbX_2_–HfY_2_	Par.	NbSe_2_–HfS_2_	NbSe_2_–HfSe_2_	NbTe_2_–HfS_2_	NbTe_2_–HfSe_2_
Stacking (a)	*E*_b_	−0.0135	−0.0141	−0.0176	−0.0264
	*d*	2.552	2.647	3.719	3.784
Stacking (b)	*E*_b_	−0.0205	−0.0217	−0.0269	−0.0513
	*d*	2.551	2.617	3.631	3.746
Stacking (c)	*E*_b_	−0.0192	−0.0204	−0.0269	−0.0267
	*d*	2.619	2.620	3.663	3.782
Stacking (d)	*E*_b_	−0.0195	−0.0206	−0.0265	−0.0269
	*d*	4.945	2.619	3.661	3.84

**Table 2:** Lattice constant (*a* in Å), bond length (in Å), elastic constants (*C*_11_, *C*_12_, *C*_13_ and *C*_66_), bulk modulus (*B* in GPa), shear modulus (*G* in GPa), Young's modulus (*E* in GPa), Poisson's ratio (*σ*), Pugh's ratio (*B*/*G*) and effective masses of electrons 
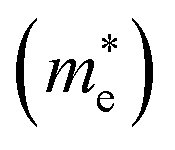
 and holes 
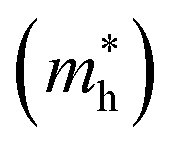
 for NbX_2_–HfY_2_ (X = Se, Te; Y = S, Se) MS vdWHs

Param.	NbSe_2_–HfS_2_	NbSe_2_–HfSe_2_	NbTe_2_–HfS_2_	NbTe_2_–HfSe_2_
*a*	3.50	3.57	3.60	3.68
Nb-X	2.62	2.63	2.88	2.89
Hf-Y	2.55	2.64	2.55	2.64
*C*_11_	69.8	77.6	57.0	61.2
*C*_12_	18.17	35.1	25.2	23.7
*C*_66_	25.8	21.2	16.19	18.75
*B*	20.35	27.7	17.19	18.3
*G*	10.67	8.25	8.13	7.20
*E*	27.23	22.5	21.07	19.1
*σ*	0.28	0.36	0.29	0.32
*B*/*G*	1.90	3.35	2.11	2.54
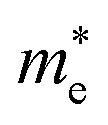	0.225	0.239	0.253	0.273
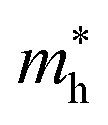	0.567	0.284	0.284	0.284

Phonon dispersion spectra (Fig. 2(a–d)) confirm the dynamic stability of the NbX_2_–HfY_2_ (X = Se, Te; Y = S, Se) vdWHs. For the six atoms in the unit cell of NbX_2_–HfY_2_ vdWHs, 3 acoustic (transverse (TA), longitudinal (LA), and out-of-plane (ZA)) and 15 optical branches of the phonon modes are comprised at the *Γ*-point. Importantly, the ZA mode near the *Γ*-point exhibits a quadratic dispersion (*ω* ∝ *q*^2^), which is the feature of the weak interlayer coupling characteristic of 2D vdW materials. The optical phonon modes show the highest frequencies for the NbSe_2_–HfS_2_ system, followed by NbTe_2_–HfS_2_, NbSe_2_–HfSe_2_ and NbTe_2_–HfSe_2_ vdWHs. This trend is primarily governed by the atomic masses of the constituent elements. These results are consistent with experimental Raman spectroscopy findings for other 2D materials and their vdWHs.^50,51^ To verify dynamic stability at the critical strains, where the Schottky-to-Ohmic transition occurs, the phonon dispersion spectra are calculated and presented in Fig. S13. For both NbSe_2_–HfSe_2_ and NbTe_2_–HfSe_2_, the phonon spectra at −4% compression and at +10% tension exhibit no imaginary frequencies, confirming dynamic stability of these systems.

**Fig. 2:**
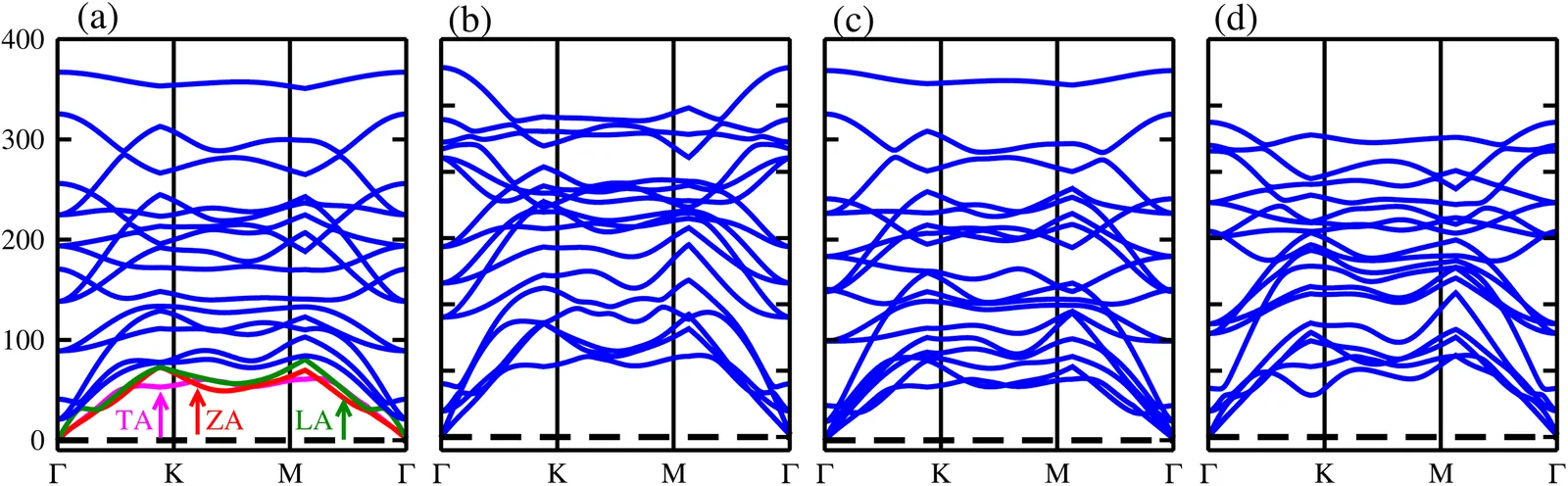
Phonon dispersion spectra of (a) NbSe_2_–HfS_2_, (b) NbSe_2_–HfSe_2_, (c) NbTe_2_–HfS_2_ and (d) NbTe_2_–HfSe_2_ MS vdWHs. The acoustic modes are color-coded for clarity: out-of-plane acoustic (ZA) in red, longitudinal acoustic (LA) in green, and transverse acoustic (TA) in magenta. The optical modes are shown in blue.

Using *Ab initio* Molecular Dynamics (AIMD)^35^ simulations at 300 K over a duration of 4 ps with a 1 fs time step, the total energy and temperature fluctuations as a function of simulation time for NbSe_2_–HfS_2_, NbSe_2_–HfSe_2_, NbTe_2_–HfS_2_, and NbTe_2_–HfSe_2_ configurations are displayed in Fig. 3. The total energy remains within a highly narrow range, with an average value nearly constant throughout the 4000-step simulation, indicating that the systems have reached thermal equilibrium without transitioning to unstable phases. Geometrical structures before and after the 300 K heating process reveal no bond breaking, significant structural distortion, or layer reconstruction. The preservation of the hexagonal lattice symmetry and the maintenance of clear interlayer spacing further confirm that these vdWHs are thermally stable at room temperature. Previous studies on similar vdWHs have also confirmed both mechanical/dynamic stability and thermal stability under ambient and moderate strain conditions,^52,53^ supporting the general expectation that structurally stable vdW systems retain their integrity under strain.

**Fig. 3:**
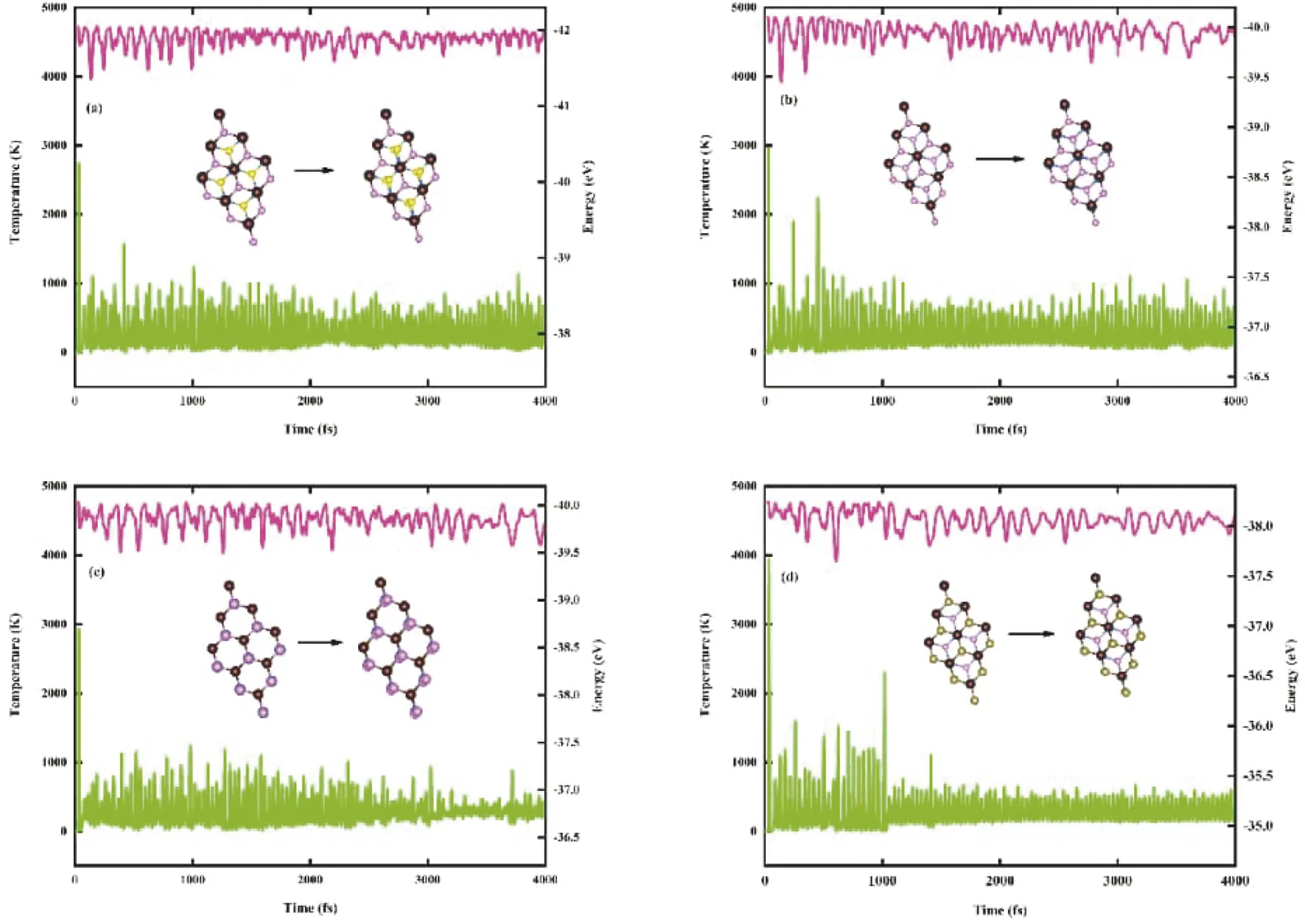
Geometrical structures before heating, with fluctuating energy and temperature and after heating of the NbX_2_–HfY_2_ (X = Se, Te; Y = S, Se) MS vdWH using AIMD simulations. The pink color represents the fluctuation of the total free energy (in eV) of the heterostructure over the simulation time. The green color represents the fluctuation of the system temperature (in K) over the simulation time.

All NbX_2_–HfY_2_ MS vdWHs satisfy the general in-plane mechanical stability criteria^54,55^ for 2D materials, *C*_11_ > 0, *C*_66_ > 0, and *C*_11_ − |*C*_12_| > 0, shown in Table 2. For instance, in the NbSe_2_–HfSe_2_ system, *C*_11_ = 77.6 GPa and *C*_12_ = 35.1 GPa, satisfying the condition 77.6 > 35.1. The non-monotonic change in the transverse elastic constant (*C*_12_) is caused by a balance between how strongly charges are pulled across the interface and the physical size of the atoms. From S to Se in the NbSe_2_ systems, the local charge trapping at the interface is reduced, making the layers more flexible sideways under stress and increasing *C*_12_. On the other hand, the larger Te and Se atomic sizes in NbTe_2_–HfSe_2_ push the layers further apart and weaken their electronic interaction, which limits this sideway flexibility and leads to a decrease in *C*_12_. The bulk modulus (*B*) ranges from 17.19 GPa for NbTe_2_–HfS_2_ to 27.7 GPa for NbSe_2_–HfSe_2_, indicating that the latter is the most resistant to hydrostatic pressure. The Young's modulus (*E*) varies between 19.1 GPa (NbTe_2_–HfSe_2_) and 27.23 GPa (NbSe_2_–HfS_2_), suggesting that the latter is the stiffest under longitudinal load. These calculations also show that the NbX_2_–HfY_2_ vdWHs exhibit a Pugh's ratio (*B*/*G*) greater than the 1.75 threshold, classifying it as a ductile material.^56^ The ductility is further supported by the Poisson's ratio (*σ* ranges from 0.28 to 0.36). Finally, the shear modulus (*G*) values ranging from 7.20 GPa for NbTe_2_–HfSe_2_ to 10.67 GPa for NbSe_2_–HfS_2_ indicate a moderate resistance to shear deformation. The combination of mechanical stability and ductile nature ensures that these MS vdWHs can undergo significant deformation without structural failure, vital for their performance in flexible nanoelectronic devices.^57^ The elastic constants at the critical strains, where the Schottky-to-Ohmic transition occurs, are also evaluated, see Table S1. Both NbSe_2_–HfSe_2_ and NbTe_2_–HfSe_2_ MS vdWHs satisfy the Born criteria, confirming mechanical stability at the transition point.

The electronic states of metallic NbX_2_ (ref. 58) overlap with the bandgap of the semiconducting HfY_2_ layer in NbX_2_–HfY_2_ (X = Se, Te; Y = S, Se) MS vdWHs which gives rise to type III (broken-gap) band alignment,^59^ presented in Fig. 4. The HfY_2_ layer exhibits a preserved bandgap of approximately 1.15 eV for HfS_2_ and 0.95 eV for HfSe_2_ within the vdWHs, indicating that key properties, like the high carrier mobility of NbX_2_ and the intrinsic bandgap of HfY_2_, are completely preserved in the NbX_2_–HfY_2_ vdWHs due to the weak vdW interaction, fitting them for potential applications in FETs.^60,61^

**Fig. 4:**
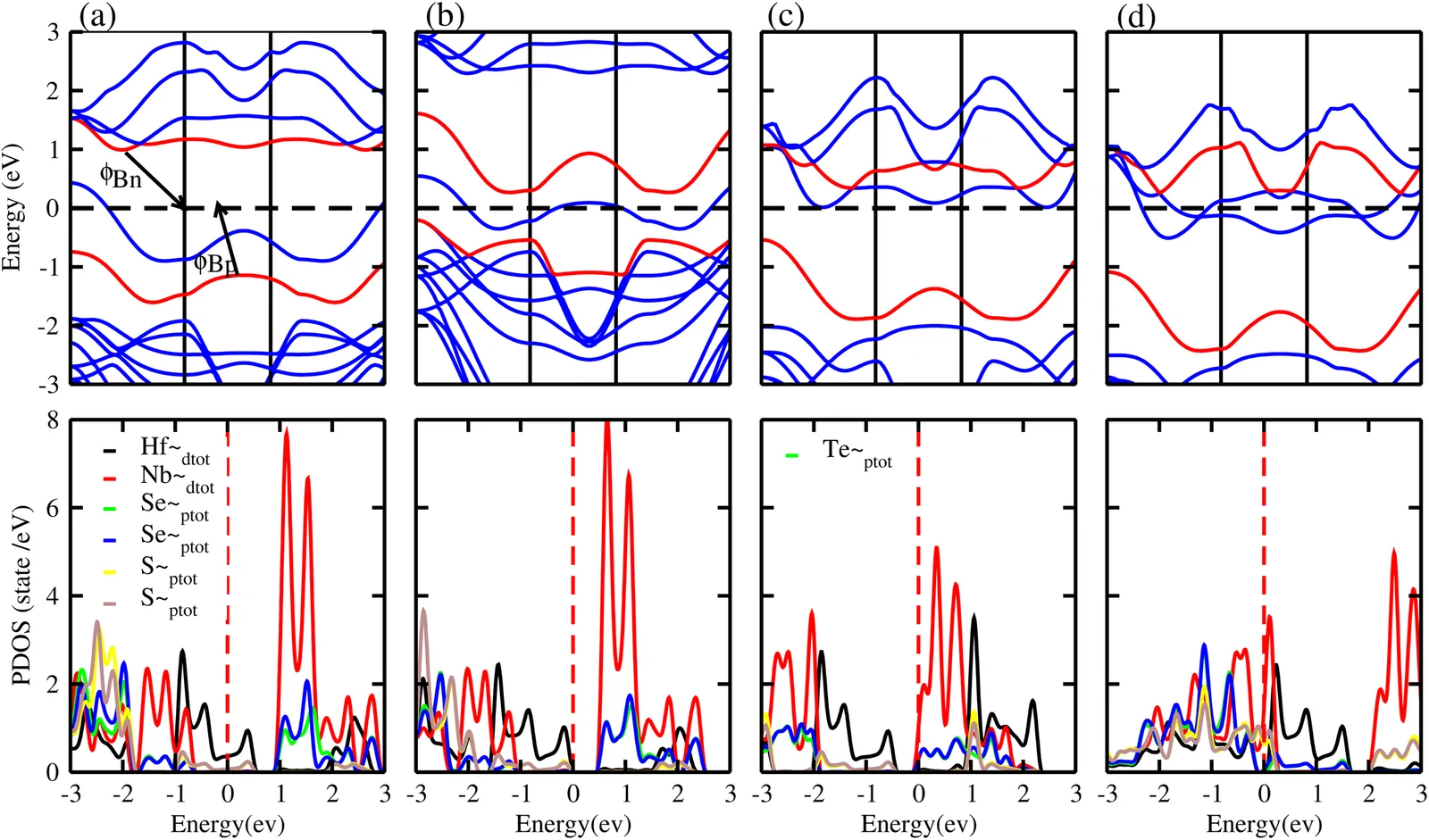
Electronic band structure (first row) and partial density of states (second row) of (a) NbSe_2_–HfS_2_ (b) NbSe_2_–HfSe_2_ (c) NbTe_2_–HfS_2_ (d) NbTe_2_–HfSe_2_ MS vdWHs. The blue color represents the electronic states of the metallic NbX_2_ (X = Se, Te) layer, while the orange color represents the electronic states of the semiconducting HfY_2_ (Y = S, Se) layer.

To validate the robustness of the PBE calculations against the well-known bandgap underestimation, additional HSE06 hybrid functional calculations are performed for all four vdWHs at equilibrium and the transition point (for NbSe_2_–HfSe_2_ and NbTe_2_–HfSe_2_ at −4% and +10% strain (Fig. S14 and S15)). At equilibrium, the HSE06 band structures preserve the type-III band alignment and n-type Schottky contact character, with bandgaps that are larger by ∼0.6–1.2 eV compared to those obtained by PBE calculations. At −4% compressive strain, the HSE06 bandgap remained finite (0.61 eV for NbSe_2_–HfSe_2_ and 0.65 eV for NbTe_2_–HfSe_2_), indicating that the Schottky-to-Ohmic transition is not confirmed at this strain at the HSE06 level. However, these values of the bandgap are significantly smaller than those at equilibrium (1.24 eV and 1.15 eV, respectively), confirming the trend of strain-induced band-gap reduction and SBH reduction. At +10% tensile strain, the systems remain n-type Schottky contacts with reduced barriers for both PBE and HSE06 type calculations. These results demonstrate that the predicted Schottky-to-Ohmic transition is qualitatively robust, though the quantitative critical strain is method-dependent and shifts to more negative values when using HSE06 due to the increased bandgap. PDOS in Fig. 4 further confirms that the states at the Fermi level (*E*_F_) are predominantly governed by the Nb-d orbitals, with a minor contribution from the X-p states of the NbX_2_ layer. The VBM and CBM of the HfY_2_ layers (that do not cross the *E*_F_) are primarily composed of Y-p and Hf-d orbitals (Y = S, Se). The correlation between PDOS evolution and SBH modulation reveals a direct microscopic mechanism. Under compressive strain, the Hf-d orbitals of the semiconducting HfY_2_ layer progressively shift toward *E*_F_ and hybridize with the Nb-d orbitals of the metallic NbX_2_ layer. This hybridization is most pronounced at the critical strains where the Schottky-to-Ohmic transition occurs: for NbSe_2_–HfSe_2_ at −4% strain, a distinct Hf-d peak emerges at *E*_F_ (Fig. S4), coinciding with the vanishing of both *Φ*_B_n__ and *Φ*_B_p__ (Table 4). Conversely, tensile strain generally shifts Hf-d states away from *E*_F_, increasing the SBH, except in NbSe_2_–HfSe_2_ at +10% where the Hf-d states cross *E*_F_ (Fig. S4(j)). The HfS_2_-based systems exhibit more robust n-type character with less dramatic Hf-d shifting, consistent with their higher SBH values under strain (Table 3), while HfSe_2_-based systems display higher sensitivity to band shifting. These observations establish strain-tunable Nb-d/Hf-d orbital hybridization as the microscopic origin of contact-type modulation in these vdWHs.

**Table 3:** Lattice constant (*a*), potential drop (Δ*V*), work function (*ϕ*), ionization energy (I), electron affinity (*χ*) and height of the Schottky barrier (n-type-*Φ*_B_n__; p-type-*Φ*_B_p__), parameter of the Fermi level pinning (*S*_FLP_), tunneling barrier (*Φ*_TB_), tunneling barrier width (*W*_TB_), and comprehensive factor (*C*) for NbX_2_-HfS_2_ (X = Se, Te) MS vdWHs

Strain	−10	−8	−6	−4	−2	0	2	4	6	8	10
**NbSe**_**2**_ **–HfS**_**2**_											
*a*	3.15	3.22	3.29	3.36	3.43	3.50	3.57	3.64	3.71	3.78	3.85
Δ*V*	1.17	1.17	1.06	0.90	0.85	3.61	0.96	0.85	0.69	0.59	0.53
*ϕ*	2.05	2.24	2.43	2.60	2.74	2.95	2.99	3.08	3.16	3.17	3.21
*I*	5.08	5.07	5.05	5.02	4.93	4.88	4.65	4.52	4.38	4.18	4.01
*χ*	4.84	4.48	4.19	3.94	3.77	3.70	3.48	3.41	3.32	3.20	3.12
*Φ*_B_n__	2.79	2.24	1.76	1.34	1.03	0.75	0.49	0.33	0.16	0.03	0.09
*Φ*_B_p__	2.93	2.75	2.58	2.42	2.19	1.93	1.66	1.44	1.22	1.01	0.80
*S*_FLP_	0.74	0.68	0.61	0.57	0.50	0.42	0.37	0.32	0.26	0.21	0.17
*Φ*_TB_	4.69	4.48	4.37	4.21	4.05	3.47	3.36	3.20	3.10	2.94	2.83
*W*_TB_	2.48	2.48	2.48	2.48	2.48	2.07	1.82	1.82	1.76	1.70	1.64
*C*	28.85	27.55	26.88	25.89	24.91	14.87	11.13	10.60	9.60	8.45	7.61

**NbTe**_**2**_ **–HfS**_**2**_											
*a*	3.24	3.31	3.38	3.45	3.52	3.60	3.67	3.74	3.81	3.88	3.96
Δ*V*	1.59	1.33	1.12	0.95	0.90	2.39	0.58	0.64	0.85	0.48	0.42
*ϕ*	0.99	1.31	1.50	1.81	1.98	2.41	2.33	2.52	2.69	2.75	2.80
*I*	5.47	5.46	5.45	5.44	5.30	5.29	4.96	4.72	4.61	4.50	4.29
*χ*	5.26	4.95	4.56	4.36	4.14	4.31	3.82	3.72	3.63	3.52	3.39
*Φ*_B_n__	4.27	3.64	3.06	2.55	2.16	1.90	1.49	1.20	0.97	0.77	0.60
*Φ*_B_p__	4.40	4.15	3.88	3.63	3.32	2.88	2.63	2.31	2.02	1.75	1.50
*S*_FLP_	1.40	1.25	1.11	1.00	0.90	0.76	0.68	0.59	0.51	0.43	0.36
*Φ*_TB_	4.59	4.48	4.32	4.16	4.00	3.79	3.58	3.47	3.29	3.15	2.99
*W*_TB_	1.66	1.67	1.70	1.71	1.73	1.92	1.64	1.58	1.54	1.52	1.49
*C*	12.50	12.49	12.48	12.16	11.97	13.97	9.63	8.66	7.80	7.28	6.64

The carrier effective masses (
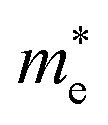
 and 
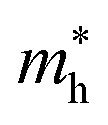
) in the NbX_2_–HfY_2_ MS vdWHs are calculated and summarized in Table 2. The relatively low effective mass across these systems suggests high carrier mobility, identifying them as promising candidates for high-speed nanoelectronic applications.^62^ At the equilibrium state, the NbSe_2_–HfS_2_ vdWH exhibits the lowest 
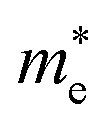
, indicating superior electron mobility and strong electronic dispersion at the conduction band edge. However, the variation in hole effective mass across the series significantly influences the predicted transport behavior. For instance, in the NbSe_2_–HfS_2_ system, the 
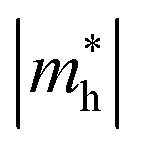
 is more than double the electron mass, suggesting a strong preference for n-type transport.^63^ In contrast, for the NbTe_2_–HfS_2_ and NbTe_2_–HfSe_2_ systems, both the 
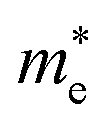
 and 
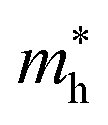
 are increasingly comparable, indicating the potential for balanced ambipolar transport, where both electrons and holes contribute effectively to the current. These results underscore the tunable nature of the transport properties in NbX_2_–HfY_2_ vdWHs through chalcogen substitution.

The electrostatic potential in Fig. 5 shows a noticeable potential difference (Δ*V*) across the interface due to charge redistribution and interface dipole formation.^64^ The calculated Δ*V* values in Tables 3 and 4 show a larger potential drop in the unstrained NbSe_2_–HfY_2_ systems, suggesting stronger interfacial charge transfer and a more pronounced interface dipole moment. This variation originates from the differences in electronegativity between the chalcogen atoms and the strength of the interlayer coupling that modulate the band alignment relative to the *E*_F_, and hence a critical factor in determining the SBH.^65^ Concurrently, the work functions (*ϕ*) of the vdWHs are 2.95 eV (NbSe_2_–HfS_2_), 3.46 eV (NbSe_2_–HfSe_2_), 2.41 eV (NbTe_2_–HfS_2_), and 2.33 eV (NbTe_2_–HfSe_2_), significantly modified compared to those of isolated monolayers of NbSe_2_ (4.43 eV) and NbTe_2_ (3.76 eV), indicating favorable conditions for carrier injection at the metal–semiconductor interface. The charge density difference (Δ*ρ*) in Fig. 5 confirms the spatial redistribution of charges. Electrons are transferred from the NbX_2_ layer to the HfY_2_ layer, characterized by electron depletion near the interfacial Nb/X atoms and accumulation around the interfacial Hf/Y atoms. The planar average electrostatic potential along the *z*-axis (Fig. 5) illustrates the potential step across the vdWH interfaces, with deep potential wells corresponding to the atomic planes of Nb, X (Se and Te), Hf, and Y (S and Se). The oscillations clearly distinguish the shallower potential region associated with the metallic NbX_2_ layer from the deeper potential region of the semiconducting HfY_2_ layer. This gradient confirms that electrons are spontaneously transferred from the metal to the semiconductor, establishing strong interlayer coupling and stable vdW interactions at the interface. Consistent with established studies on 2D interfaces,^66^ this charge transportation generates an internal electric field,^67^ that modulates the effective carrier concentration and facilitates the fine-tuning of the *ϕ*, essential for optimizing device performance. Bader charge analysis^68^ reveals that the metallic (semiconducting) NbX_2_(HfY_2_) layers act as charge donors (acceptors). Specifically, a charge transfer is observed from NbSe_2_ to HfS_2_, of −0.021e in NbSe_2_–HfSe_2_, of −0.06e in NbTe_2_–HfS_2_ and of −0.042e in NbTe_2_–HfSe_2_ vdWHs, see Table 5. The variations in the magnitude of charge transfer are consistent with the electronegativity differences between the constituent chalcogen atoms. Ultimately, this electronic interaction drives the formation of the Schottky contact^69^ and Fermi level pinning (FLP),^70^ confirming that these vdWHs are not merely physical stacks but electronically coupled systems with significant potential for use in nanoelectronic applications.^71^ The Δ*V* across the unstrained NbX_2_–HfY_2_ interfaces exhibits a highly consistent trend that is fundamentally governed by the ϕ mismatch and structural parameters. The NbSe_2_-based systems display significantly larger potential drop (3.61–3.77 eV) compared to the NbTe_2_-based counterparts (1.81–2.39 eV), attributed to the larger *ϕ* of isolated NbSe_2_ that drives a more substantial spontaneous charge transfer to align the *E*_F_. Additionally, the smaller atomic radius of Se than Te leads to a highly compact interlayer distance (*d* ≈ 2.55 Å *vs.* 3.63 Å), enabling stronger orbital overlap and a more localized interface dipole that sustains a much larger electrostatic potential step across the vdW gap. The formation of the NbX_2_–HfY_2_ (X = Se, Te; Y = S, Se) MS vdWHs induces a charge transfer, creating an interfacial dipole moment, that tunes the position of the VBM and CBM relative to the *E*_F_ and sets a Schottky contact at the interface,^69^ see Fig. 4. Effective control over carrier injection efficiency in MS vdWHs is directly governed by the Schottky barrier height (SBH) (SBH is assessed using the Schottky–Mott (SM) rule^41,42^ against first-principles calculations^40^), shown in Fig. 6(a–d), where the SM model is a classical, idealized framework that treats the metallic and semiconducting components as non-interacting entities and DFT calculations explicitly account for these non-ideal interactions by modeling the entire heterostructure as a single, combined system.^72–74^ These calculations confirm an n-type (*Φ*_B_n__ < *Φ*_B_p__) Schottky contact, favoring electron conduction instead of holes, a feature typically associated with enhanced carrier transport in n-type channel devices.^75^ Lowering the contact resistance, weaken the potential energy barriers, facilitating carriers transport and enhance the current flow while minimizes current leakage on the other side increasing the contact resistance strengthen the potential energy barrier.^76^ Therefore, the impact of strain engineering on the electronic band structure of these systems (see Fig. S1, S3, S5 and S7) is investigated to explore the possibility of achieving Ohmic contact. This phenomenon mirrors recent observations in other vdWHs,^76^ where external strain serves as an effective tool for modulating the SBH and contact properties.^77^ In the case of the NbSe_2_–HfS_2_ MS contact, compressive strain significantly increases the barriers; at −10% strain, the calculated p(n)-type barriers *Φ*_B_p__ (*Φ*_B_n__) reach 2.93 (2.79) eV. In contrast, the SM rule predicts much lower values of *Φ*_B_p__ (*Φ*_B_n__) = 1.00 (0.86) eV at the same compressive level, as shown in Fig. 6(a) and Table 3. Conversely, the application of maximum tensile strain (10%) leads to a systematic decrease (increase) in the hole (electron) barrier, resulting in *Φ*_B_p__ (*Φ*_B_n__) = 0.80 (0.09) eV. Under this same tensile limit, the SM rule predicts significantly different values of *Φ*_B_p__ (*Φ*_B_n__) = 0.77 (1.66) eV. These discrepancies clearly demonstrate that the real contact behavior deviates from the simple SM rule due to the combined influence of strain-induced electronic shifts and interface dipole effects. In the case of the NbTe_2_–HfS_2_ MS contact, compressive strain significantly increases the p-type (n-type) barrier, reaching *Φ*_B_p__ (*Φ*_B_n__) = 4.40 (4.27) eV at −10% strain. For the same compressive level, the SM rule predicts much lower magnitudes of *Φ*_B_p__ (*Φ*_B_n__) = 2.24 (2.11) eV. Conversely, the application of 10% tensile strain leads to a systematic decrease in the hole barrier and an increase in the electron barrier, reaching a minimum value of *Φ*_B_p__ (*Φ*_B_n__) = 1.50 (0.60) eV. Under this same tensile limit, the SM rule predicts values of *Φ*_B_p__ (*Φ*_B_n__) = 0.15 (0.75) eV, demonstrating a notable discrepancy between both results. For NbSe_2_–HfSe_2_ MS vdWHs, compressive strain initially leads to high barrier heights; at −2% strain, the calculated values are *Φ*_B_p__ = 2.13 eV and *Φ*_B_n__ = 1.88 eV. At this same compressive level, the SM rule predicts significantly lower values of *Φ*_B_p__ (*Φ*_B_n__) = 0.11 (0.14) eV. When the compressive strain is increased further to −4%, the system reaches a metallic state and the heterostructure becomes Ohmic (zero barrier), see Table 3 and Fig. 6(b). Conversely, tensile strain results in a systematic reduction of the hole barrier and an increase (less negative/approaching positive) in the electron barrier, reaching *Φ*_B_p__ (*Φ*_B_n__) = 1.20 (0.28) eV at 10% strain. Under this same tensile limit, the SM rule predicts *Φ*_B_p__ (*Φ*_B_n__) = −0.57 (1.49) eV, again highlighting the failure of the Schottky–Mott limit at these strained vdW interfaces. For the NbTe_2_–HfSe_2_ MS vdWHs, compressive strain leads to a steady increase in both Schottky barriers. At −2% strain, the calculated values reach *Φ*_B_p__ = 3.16 eV and *Φ*_B_n__ = −2.94 eV. Under this same compressive strain, the SM rule predicts lower magnitudes for *Φ*_B_p__ (*Φ*_B_n__) of 1.36 (−1.14) eV. When the compressive strain is further increased to −4%, the barriers effectively vanish as the system reaches a metallic state, making the heterostructure Ohmic. Detailed values are provided in Table 3 and Fig. 6(d). Conversely, tensile strain reduces both the n- and p-type barriers, with the hole barrier reaching a minimum value of *Φ*_B_p__ = 1.91 eV and *Φ*_B_n__ of 0.99 eV at 10% strain. Under this maximum tensile limit, the SM rule predicts values of *Φ*_B_p__ (*Φ*_B_n__) = 0.50 (0.42) eV. These findings demonstrate that the SM rule consistently underestimates the actual barrier heights across the NbX_2_–HfY_2_ series, often by more than 1.0 eV. This significant discrepancy indicates a strong FLP effect at the MS vdWH interfaces.^78^ The traditional SM model fails to account for the complex interfacial dipoles and the redistribution of electronic states, such as metal-induced gap states (MIGSs) that arise during contact formation. Such microscopic interactions significantly modify the potential alignment, shifting the band edges relative to the *E*_F_ and resulting in the higher barrier heights observed in these calculations. This extreme sensitivity in the wide modulation range, combined with the shift in carrier-favored transport, makes NbX_2_–HfY_2_ (X = Se, Te; Y = S, Se) MS vdWHs potential candidates for strain-controlled, multifunctional electronic devices.^79^ The Schottky-to-Ohmic transition observed in NbX_2_–HfY_2_ vdWHs under external strain arises from a synergistic yet hierarchically ordered set of mechanisms. The primary driver is the strain-induced shift of the CBM and VBM of the HfY_2_ semiconductor relative to the *E*_F_ of the metallic NbX_2_ layer. As shown in Tables 3, 4 and Fig. S1–S8, the convergence of the band edges with *E*_F_ under compressive strain (*e.g.*, at −4% for NbSe_2_–HfSe_2_ and NbTe_2_–HfSe_2_) directly eliminates the Schottky barrier, yielding an Ohmic contact. This band-edge modulation is accompanied by strain-tunable interfacial charge redistribution, which alters the interface dipole and electrostatic potential drop (Fig. 5 and Tables 3, 4), providing a secondary contribution to the SBH modulation. Additionally, strain reduces both the tunneling barrier height (Φ_TB_) and width (*W*_TB_), as reflected in the comprehensive factor *C* (Tables 3 and 4), thereby facilitating carrier injection even before the SBH completely vanishes. At the microscopic level, these effects originate from enhanced hybridization between Nb-d and Hf-d orbitals near *E*_F_ under strain, as evidenced by the PDOS analysis (Fig. S2, S4, S6, and S8). Collectively, these mechanisms establish strain engineering as a powerful strategy for achieving low-resistance contacts in 2D nanoelectronic devices. The convergence of these barriers at low energy levels suggests that NbX_2_–HfY_2_ vdWHs can function as efficient contacts facilitating the injection of both electrons and holes, though the consistently lower *Φ*_B_n__ values across all strain states indicate a preference for n-type transport. Specifically, under compressive strain, the NbX_2_–HfSe_2_ vdWHs exhibit a clear transition from Schottky to Ohmic contact as the CBM crosses the *E*_F_ at −4% strain. This vanishing of the n-type Schottky barrier (*Φ*_B_n__ ≤ 0) for electron injection results in a direct Ohmic transport mechanism that significantly reduces contact resistance and enhances n-type carrier injection efficiency.^80^ The degree of the FLP^81^ induced by the interface dipole (ID) is analyzed *via* the slope parameter *S*.^82,83^ The computed *S*_FLP_ values for NbX_2_–HfY_2_ (X = Se, Te; Y = S, Se) MS vdWHs exhibit a significant correlation with lattice modulation. Specifically, compressive strain systematically increases the *S*_FLP_, reaching peak values of 0.74 eV for NbSe_2_–HfS_2_ and 1.40 eV for NbTe_2_–HfS_2_ at −10% strain. This behavior physically corresponds to a deepening of the Schottky barrier and a widening of the energy offset between the metal *E*_F_ and the semiconductor band edges. Conversely, the application of tensile strain facilitates a pronounced reduction in *S*_FLP_ across all studied interfaces. For instance, at 10% tensile strain, the *S*_FLP_ decreases to 0.17 eV and 0.36 eV for the HfS_2_-based interfaces (NbSe_2_ and NbTe_2_, respectively). A similar trend is observed for the HfSe_2_-based systems, where the *S*_FLP_ drops to 0.25 eV (NbSe_2_–HfSe_2_) and 0.48 eV (NbTe_2_–HfSe_2_) at 10% strain. Most notably, the HfSe_2_-series demonstrates a unique transition at −4% compressive strain, where the *S*_FLP_ vanishes entirely (0 eV). This strain-dependent *S*_FLP_ variation exhibits a quantitative correlation with both interfacial charge redistribution (Table 5) and Δ*V* (Tables 3 and 4). Under compressive strain, enhanced charge transfer (*e.g.*, NbTe_2_–HfS_2_: −0.060e) strengthens the interface dipole, increasing *S*_FLP_ (weaker pinning), while tensile strain reduces charge transfer, decreasing *S*_FLP_ (stronger pinning). At the critical −4% strain for HfSe_2_-based systems, the *S*_FLP_ vanishes entirely (0 eV), coinciding with the merger of the semiconductor band edges with *E*_F_ and the transition to Ohmic contact. However, the correlation between Δ*V* and *S*_FLP_ is system-dependent: at small compressive strains (*e.g.*, −2%), *S*_FLP_ generally increases due to enhanced orbital hybridization even when Δ*V* decreases (*e.g.*, NbSe_2_–HfS_2_: Δ*V* 3.61 → 0.85 eV and *S*_FLP_: 0.42 → 0.50; NbTe_2_–HfS_2_: Δ*V* 2.39 → 0.90 eV, *S*_FLP_ is 0.76 → 0.90), indicating that *S*_FLP_ is primarily governed by the electronic structure (hybridization, MIGSs) at the interface rather than the macroscopic electrostatic potential alone. The exception is NbSe_2_–HfSe_2_, where weak charge transfer (−0.021e, Table 5) prevents sufficient hybridization, leading to a decrease in both Δ*V* (3.77 → 0.96 eV) and *S*_FLP_ (0.56 → 0.48) at −2% strain. These results suggest that while tensile strain effectively pulls the *E*_F_ closer to the conduction band to minimize the barrier, specific compressive points in HfSe_2_ can trigger a transition from a Schottky contact to a high-efficiency Ohmic regime.

**Fig. 5:**
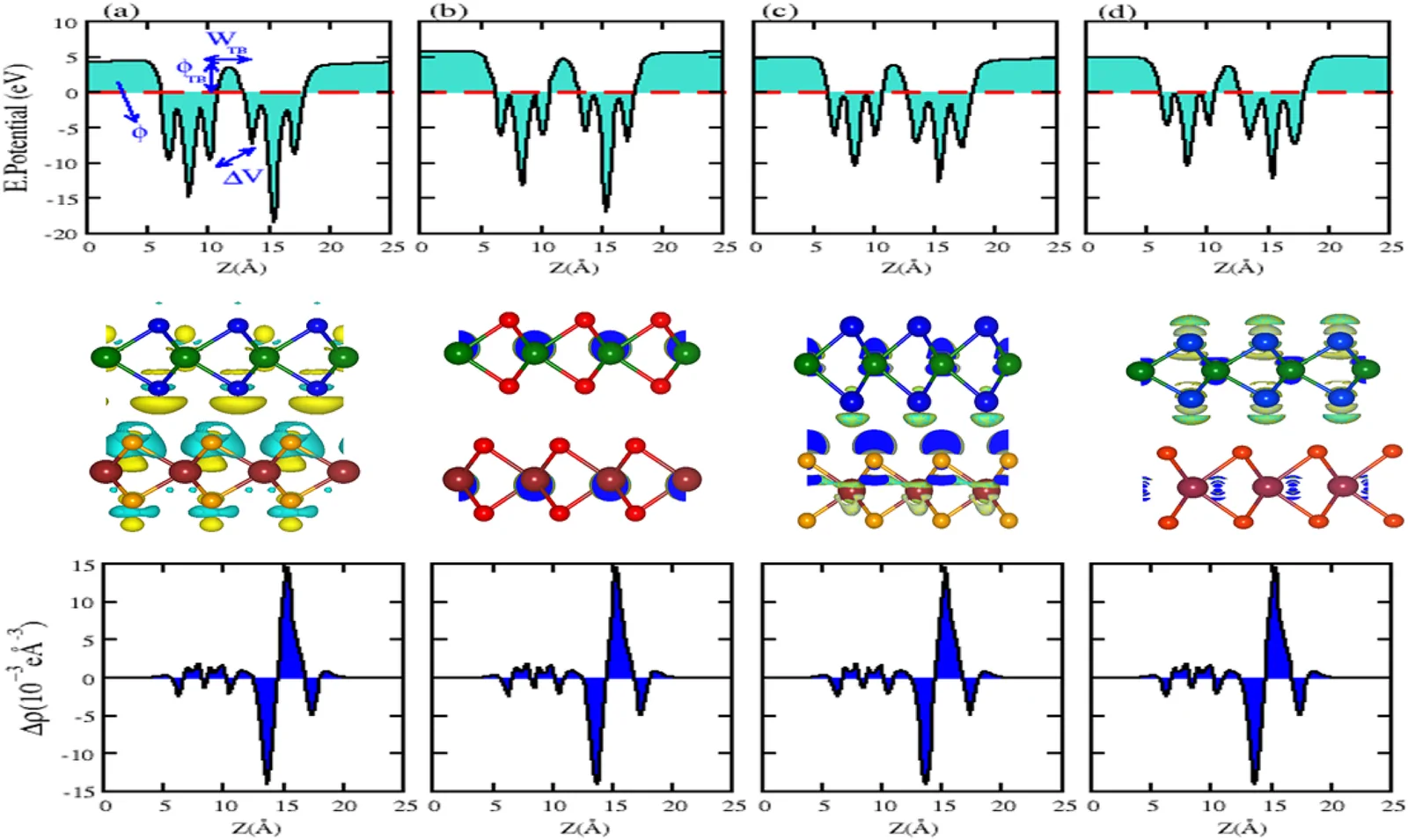
Electrostatic potential (first row), charge density difference (second row), and planar average electrostatic potential along the *z*-axis (third row) of (a) NbSe_2_–HfS_2_ (b) NbSe_2_–HfSe_2_ (c), NbTe_2_–HfS_2_ (d), and NbTe_2_–HfSe_2_ MS vdWHs.

**Table 4:** Lattice constant (*a*), potential drop (Δ*V*), work function (*ϕ*), ionization energy (*I*), electron affinity (*χ*) and height of the Schottky barrier (n-type-*Φ*_B_n__; p-type-*Φ*_B_p__), parameter of the Fermi level pinning (*S*_FLP_), tunneling barrier (*Φ*_TB_), tunneling barrier width (*W*_TB_), and comprehensive factor (*C*) for NbX_2_–HfSe_2_ (X = Se, Te) MS vdWHs

Strain	−4	−2	0	2	4	6	8	10
**NbSe**_**2**_ **–HfSe**_**2**_								
*a*	3.42	3.49	3.57	3.64	3.71	3.78	3.85	3.92
Δ*V*	1.11	0.96	3.77	1.11	0.90	0.69	0.74	0.80
*ϕ*	2.21	2.38	3.46	2.70	2.79	2.89	2.92	2.97
*I*	4.59	4.51	5.94	4.56	4.54	4.48	4.30	4.17
*χ*	4.56	4.26	5.41	3.80	3.60	3.48	3.34	3.25
*Φ*_B_n__	0	1.88	1.95	1.05	0.82	0.59	0.42	0.28
*Φ*_B_p__	0	2.13	2.48	1.85	1.76	1.59	1.38	1.20
*S*_FLP_	0	0.48	0.56	0.41	0.38	0.34	0.29	0.25
*Φ*_TB_	4.27	4.11	4.69	3.47	3.36	3.20	3.10	2.94
*W*_TB_	2.54	2.54	2.41	1.98	1.98	1.92	1.89	1.89
*C*	27.55	26.52	27.24	13.60	13.17	11.80	11.07	10.50

**NbTe**_**2**_ **–HfSe**_**2**_								
*a*	3.53	3.60	3.68	3.75	3.82	3.90	3.97	4.04
Δ*V*	1.17	1.06	1.81	0.49	0.51	0.53	0.58	0.69
*ϕ*	1.41	1.70	2.33	2.08	2.22	2.33	2.46	2.58
*I*	4.96	4.85	5.20	4.87	4.86	4.73	4.61	4.49
*χ*	4.92	4.63	4.67	4.10	3.92	3.74	3.65	3.57
*Φ*_B_n__	0	2.94	2.34	2.02	1.70	1.41	1.19	0.99
*Φ*_B_p__	0	3.16	2.87	2.79	2.64	2.40	2.15	1.91
*S*_FLP_	0	0.91	0.79	0.75	0.69	0.62	0.54	0.48
*Φ*_TB_	4.32	4.11	3.63	3.68	3.47	3.36	3.20	3.05
*W*_TB_	2.02	1.95	1.98	1.83	1.80	1.80	1.77	1.70
*C*	17.63	15.63	14.23	12.32	11.24	10.89	10.03	8.81

**Table 5:** Redistribution of charges at the interface of NbX_2_–HfY_2_ (X = Se, Te; Y = S, Se) vdWHs

TaTe_2_–MS_2_	NbSe_2_–HfS_2_	NbSe_2_–HfSe_2_	NbTe_2_–HfS_2_	NbTe_2_–HfSe_2_
NbX_2_	−0.07e^−^	−0.021e^−^	−0.060e^−^	−0.042e^−^
HfY_2_	0.071h^+^	0.022h^+^	0.065h^+^	0.042h^+^

**Fig. 6:**
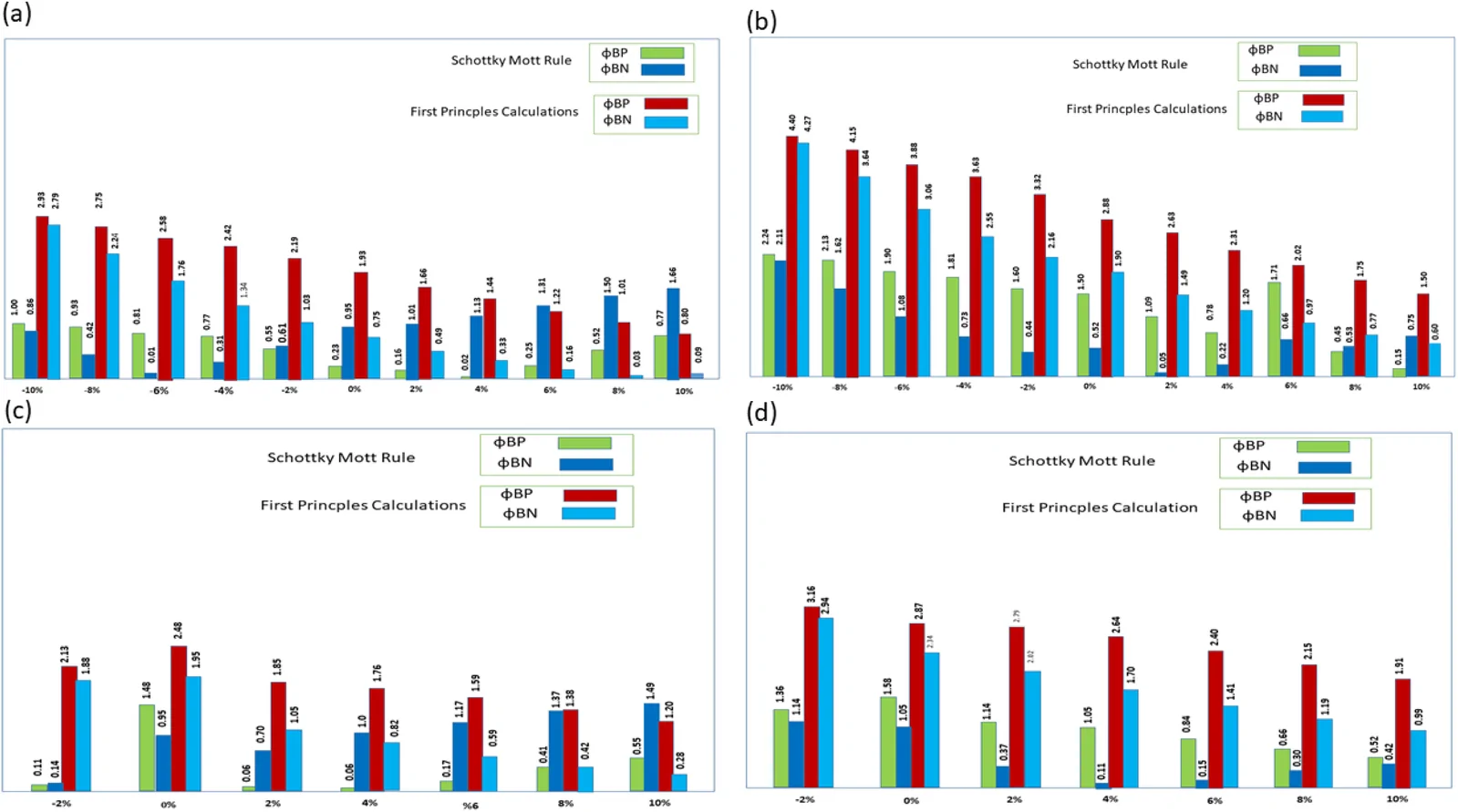
Strain engineering of the calculated Schottky barrier height for (a) NbSe_2_–HfS_2_, (b) NbTe_2_–HfS_2_, (c) NbSe_2_–HfSe_2_, and (d) NbTe_2_–HfSe_2_ MS vdWHs.

Following our previous discussion, strain engineering modulates both SBHs converting a Schottky to Ohmic contact. It also changes the overlapping of orbitals and hence initiates a tunneling barrier (TB) at the interface of NbX_2_–HfY_2_ (X = Se, Te; Y = S, Se) MS vdWHs.^84^ Using the unstrained electrostatic potential profile in Fig. 6, the calculated *Φ*_TB_ (*W*_TB_) for NbSe_2_–HfS_2_ is 3.47 eV (2.07 Å), for NbSe_2_–HfSe_2_ is 4.69 eV (2.41 Å), for NbTe_2_–HfS_2_ is 3.79 eV (1.92 Å), and for NbTe_2_–HfSe_2_ is 3.63 eV (1.98 Å) with a *P*_TB_ of 0.59%, 0.55% and 0.70% respectively. The modulated electrostatic potentials are shown in SI Fig. S9–S12. In the case of NbSe_2_–HfS_2_ MS vdWHs, tensile strain decreases the comprehensive factor (*C*) from 14.87 to 7.61, while compressive strain increases this factor to 28.85. This variation is based on tuning of *Φ*_TB_ (*W*_TB_) under strain engineering. Both the *Φ*_TB_ (*W*_TB_) values decrease from 3.47 eV (2.07 Å) to 2.83 eV (1.64 Å) under tensile strain and increase to 4.69 eV (2.48 Å) with compressive strain, see Table 3. In the case of NbSe_2_–HfSe_2_ MS vdWHs, tensile strain decreases the *C* factor from 27.24 to 10.50, while compressive strain (at −4%) increases this factor to 27.55. This variation is based on tuning of *Φ*_TB_ (*W*_TB_) under strain. Both the *Φ*_TB_ (*W*_TB_) values decrease from 4.69 eV (2.41 Å) to 2.94 eV (1.89 Å) under tensile strain and increase to 4.27 eV (2.54 Å) with compressive strain, see Table 3.^85^ In the case of NbTe_2_–HfS_2_ MS vdWHs, tensile strain decreases the *C* factor from 13.97 to 6.64, while compressive strain increases this factor to 12.50. Both the *Φ*_TB_ (*W*_TB_) values decrease from 3.79 eV (1.92 Å) to 2.99 eV (1.49 Å) under tensile strain and increase to 4.59 eV (1.66 Å) with compressive strain, see Table 3. In the case of NbTe_2_–HfSe_2_ MS vdWHs, tensile strain decreases the *C* factor from 14.23 to 8.81, while compressive strain (at −4%) increases this factor to 17.63. Both the *Φ*_TB_ (*W*_TB_) values decrease from 3.63 eV (1.98 Å) to 3.05 eV (1.70 Å) under tensile strain and increase to 4.32 eV (2.02 Å) with compressive strain, see Table 3. These results demonstrate that the effect of strain is much stronger on *Φ*_TB_, *W*_TB_ and *C*, highlighting its critical role in modulating the interfacial transport properties, enhancing the potential functionality of NbX_2_–HfY_2_ (X = Se, Te; Y = S, Se) MS vdWHs in high performance nanoelectronic devices.^65^ However, direct calculation of absolute contact resistance (in Ωµm) requires transport frameworks such as the Landauer–Buttiker formalism or NEGF methods. These DFT calculations provide quantitative indicators that are directly proportional to contact resistance. The comprehensive factor *C*, which characterizes the effective tunneling barrier, decreases significantly under tensile strain: from 14.87 to 7.61 (NbSe_2_–HfS_2_), 13.97 to 6.64 (NbTe_2_–HfS_2_), 27.24 to 10.50 (NbSe_2_–HfSe_2_), and 14.23 to 8.81 (NbTe_2_–HfSe_2_), corresponding to reductions of 49%, 52%, 61%, and 38%, respectively. Concurrently, the n-type SBH (*Φ*_B_n__) decreases by up to 88% (NbSe_2_–HfS_2_) and 68% (NbTe_2_–HfS_2_) under tensile strain. Most significantly, in the HfSe_2_-based systems, the SBH vanishes entirely at −4% compressive strain (*Φ*_B_n__ = 0 eV), while the *C* values at this critical strain are 27.55 (NbSe_2_–HfSe_2_) and 17.63 (NbTe_2_–HfSe_2_), indicating a complete Schottky-to-Ohmic transition and the elimination of the contact resistance barrier. These quantitative reductions in SBH and tunneling barriers provide a robust theoretical basis for predicting substantial contact resistance reduction in these vdWHs, consistent with experimental observations where SBH reductions of ∼1.0 eV correspond to orders-of-magnitude decreases in contact resistance.^72,74,76,78,86^

## 4. Conclusion

In summary, we performed a comprehensive first-principles investigation into the structural, mechanical, and electronic properties of NbX_2_–HfY_2_ (X = Se, Te; Y = S, Se) MS vdWHs. Binding energies, interlayer distances, and phonon dispersion spectra confirm that these interfaces are thermodynamically, dynamically, and mechanically stable. Crucially, AIMD simulations further validate the thermal stability of these systems at 300 K, demonstrating that the structural integrity of the interfaces is preserved without reconstruction or bond breakage under ambient conditions.

The weak vdW coupling ensures that the individual monolayers retain their intrinsic electronic identities specifically the high metallic conductivity of the NbX_2_ layer and the semiconducting gap of HfY_2_. At the interface, the inherent difference in *ϕ* triggers a robust charge redistribution and the formation of a significant interfacial dipole. This dipole modulates the effective *ϕ*, shifting the systems into a predominantly n-type Schottky contact regime at equilibrium.

Perhaps most significantly, these interfaces are found to be highly responsive to mechanical deformation. By applying external strain, a controlled reduction in SBH is achieved, leading to a complete Schottky-to-Ohmic transition. For HfS_2_-based systems, the n-type character remains remarkably robust, whereas in the HfSe_2_ system, strain triggers a more complex bidirectional shifting of band edges. PDOS analysis reveals that these electronic transitions are governed by the hybridization of Nb-d and Hf-d orbitals near the *E*_F_, with strain acting as a catalyst for enhanced interfacial coupling.

Given their electronic versatility, thermal robustness, and the ability to eliminate contact resistance through strain engineering, NbX_2_–HfY_2_ vdWHs emerge as ideal candidates for high-performance, strain-tunable electronics. These findings provide a theoretical roadmap for designing low-resistance 2D contacts that can effectively overcome the traditional limitations of FLP in next-generation field-effect transistors and optoelectronic sensors.

## Author contributions

Johar Ali, investigation, writing – original draft; Sahibzada I. H. Bacha, data curation and methodology; Muhammad Bila, conceptualization, validation; Bin Amin, supervision, formal analysis, writing – review & editing.

## Conflicts of interest

The author(s) declare(s) that they have no competing financial interests.

## Supplementary Material

NA-OLF-D6NA00658B-s001

## Data Availability

The data supporting this article have been included as part of the supplementary information (SI). Supplementary information is available. See DOI: https://doi.org/10.1039/d6na00658b.
