# Supplementary material for: Contact modulation in NbX2–HfY2 (X = Se, Te; Y = S, Se) van der Waals heterostructures: Schottky-to-Ohmic transition
Source: Nanoscale Adv. 2026 Sep 10. Online ahead of print. doi: 10.1039/d6na00658b (PMC13635990; doi:10.1039/d6na00658b)
Supplement: Supplementary file 1 [file NA-OLF-D6NA00658B-s001.pdf]

Supplementary Information (SI) for Nanoscale Advances.
This journal is © The Royal Society of Chemistry 2026

Supplementary Informations

Contact Modulation in NbX_2–HfY_2 (X= Se, Te; Y = S, Se) van der Waals Heterostructures: Schottky-to-Ohmic Transition

Johar Ali[1], Sahibzada I. H. Bacha[2], M. Bilal[1], B. Amin[1*]

[1]*Department of Physics, Abbottabad University of Science & Technology, Abbottabad 22010, Pakistan,* [2]*Department of Physics, Government Post-Graduate College, Mardan 23200, Pakistan*

[*] binukhn@gmail.com; +92-333-943-665

Strain engineering of the electronic band structures of the $NbSe_2$-HfS_2 vdWHs, in Figure S1, show that the system predominantly exhibits n-type behavior under both compression and tension. Both the CBM and VBM move towards the E_F under compression, leading to a decrease in the SBH and a reduction in contact resistance. At the highest compressive strain (-10%), the VBM is positioned significantly closer to E_F compared to its position in the unstrained state. However, while the VBM appears nearer to the E_F than the CBM. The calculated SBH reveal that $\Phi_{B_n} < \Phi_{B_p}$, ensuring the contact maintains its n-type character. Similarly, under tensile strain, both the CBM and VBM shift toward the E_F, further facilitating carrier injection. At the maximum tension (10%), the VBM again shifts noticeably closer to E_F than the CBM. This proximity indicates a strong modulation of the band alignment and a potential for enhanced hole-side interaction, yet the persistent relationship of $\Phi_{B_n} < \Phi_{B_p}$ confirms that the system avoids a full transition to p-type behavior, remaining a robust n-type contact across all strained configurations. PDOS of the $NbSe_2$-HfS_2 vdWHs, in Figure S2, reveals a distinct evolution of electronic states near E_F through strain engineering. The metallic character is predominantly governed by the $NbSe_2$ layer, where the Nb-d orbitals and Se-p orbitals exhibit a significant and persistent contribution at E_F. In contrast, the HfS_2 layer, characterized by Hf-d and S-p orbitals, shows a sensitive response to mechanical deformation. Under compressive strain ranging from -2% to -10% (Figure S2(a-e)), these states shift progressively toward the E_F. Specifically, at higher compression levels (-8% and -10%), the Hf-d states significantly overlap with E_F, enhancing the hybridization between the two layers. Conversely, under tensile strain from 2% to 10% (Figure S2(f-j)), the HfS_2 states gradually move away from E_F, reducing their contribution to the electronic density at the E_F and reinforcing the system's n-type semiconducting characteristics.

In case of $NbSe_2$-$HfSe_2$ vdWHs, both CBM and VBM shift toward the E_F through compressive strain. The CBM is positioned very close to E_F at -4% compression, while VBM begins to cross the E_F, signifying the onset of a metallic transition originating from the valence states. As the compression increases to -6%, the CBM also touches E_F, and by -8%, it completely crosses the E_F, indicating a fully metallized state where both electron and hole channels contribute to conduction. In contrast, under tensile strain, the CBM remains relatively stable, while the VBM progressively shifts toward the E_F. This behavior facilitates carrier injection by narrowing the hole Schottky barrier. At the maximum tensile strain of 10%, the VBM shifts significantly closer to E_F than the CBM, suggests that the

system is becoming more p-type in nature, as the energy gap for hole injection becomes smaller than that for electrons. Similar to the S-based system, the metallic character is primarily sustained by the $NbSe_2$ layer, with the Nb-d orbitals and the associated Se-p orbitals maintaining a prominent presence at E_F, illustrated in Figure S4. Under compressive strain from -2% to -10% (Figure S4(a-e)), the Hf-d states move progressively into the valence band region near E_F, with notable peaks appearing just below the E_F at higher compression. However, the most striking behavior occurs under tensile strain from 2% to 10% (Figure S4(f-j)). In this regime, the Hf-d states shift significantly toward E_F, eventually crossing it as the strain increases to 10% (Figure S4(j)), where a distinct Hf peak emerges at the E_F. This indicates a strain-induced enhancement of the metallic character and increased interlayer hybridization under high tension for the $NbSe_2$-$HfSe_2$ vdWHs.

For $NbTe_2$-HfS_2 MS vdWH, in Figure S5, both the CBM and VBM move towards the E_F, leading to a decrease in the SBH and a reduction in contact resistance through compressive strain. At the highest compressive strain (-10%), the VBM is positioned significantly closer to E_F compared to its position in the unstrained state. However, while the VBM appears nearer to the E_F than CBM in the band plots, the calculated SBH reveal that Φ_{B_n} remains lower than Φ_{B_p}, ensuring the contact maintains its n-type character. Under tensile strain (2% - 4%), both the CBM and VBM shift toward the E_F, further facilitating carrier injection. And at 6% strain both the CBM and VBM becomes very close to the E_F. But from 8% - 10% strain, both the CBM and VBM shift away from the E_F, indicating a widening of the energy gap relative to the E_F. This shift suggests that the system is moving away from a near-metallic or highly conductive state and is instead reverting toward a more semiconducting behavior, that would increase the energy barriers required for charge carriers to enter the system. The metallic nature of the heterostructure is primarily maintained by the $NbTe_2$ layer, where Nb-d orbitals and Te-p orbitals provide the dominant contribution at E_F across all strain values, see Figure S6. The HfS_2 layer, characterized by Hf-d and S-p orbitals, exhibits a high degree of tunability. Under compressive strain from -2% to -10% (Figure S6(a-e)), the Hf-d states shift significantly toward the E_F. At higher compression levels, specifically -8% (d) and -10% (e), a substantial Hf-d peak emerges directly at E_F, indicating strong interlayer hybridization and an increased contribution of the HfS_2 layer to the system's metallic conductivity. Conversely, under tensile strain from 2% to 10% (Figure S6(f-j)), the Hf-d states generally reside deeper in the valence band or higher in the conduction band,

though they remain closer to E_F than in the unstrained sulfide systems, suggesting that the $NbTe_2$ integration maintains a more integrated electronic profile even under tension.

Both the CBM and VBM move towards the E_F of the $NbTe_2$-HfS_2 vdWHs, leading to a decrease in the SBH and a reduction in contact resistance, see Figure S5. At the highest compressive strain (-10%), the VBM is positioned significantly closer to E_F compared to its position in the unstrained state. However, while the VBM appears nearer to the E_F than the CBM in the band plots, the calculated SBH reveal that $\Phi_{B_n} < \Phi_{B_p}$, ensuring the contact maintains its n-type character. Under tensile strain (2% - 4%), both the CBM and VBM shift toward the E_F, further facilitating carrier injection. But from 8% - 10% strain, both the CBM and VBM shift away from the E_F, indicating a widening of the energy gap relative to the E_F. This shift suggests that the system is moving away from a near-metallic or highly conductive state and is instead reverting toward a more semiconducting behavior The PDOS in Figure S6, demonstrates that the metallic nature of the heterostructure is primarily maintained by the $NbTe_2$ layer, where Nb-d orbitals and Te-p orbitals provide the dominant contribution at E_F across all strain values. The HfS_2 layer exhibits a high degree of tunability. Under compressive strain from -2% to -10% (Figure S6(a-e)), the Hf-d states shift significantly toward the E_F. At higher compression levels, specifically -8% (d) and -10% (e), a substantial Hf-d peak emerges directly at E_F, indicating strong interlayer hybridization and an increased contribution of the HfS_2 layer to the system's metallic conductivity. Conversely, under tensile strain from 2% to 10% (Figure S6(f-j)), the Hf-d states generally reside deeper in the valence band or higher in the conduction band, though they remain closer to E_F than in the unstrained S systems, suggesting that the $NbTe_2$ integration maintains a more integrated electronic profile even under tension.

Para.	$NbSe_2$-HfS_2		$NbSe_2$-$HfSe_2$		$NbTe_2$-HfS_2		$NbTe_2$-$HfSe_2$	
	-10%	+10%	-4%	+10%	-10%	+10%	-4%	+10%
C_{11} (GPa)	50.54	54.59	69.03	49.79	48.43	39.80	53.28	36.38
C_{12} (GPa)	49.40	12.26	28.30	10.21	42.81	6.22	25.60	4.28
C_{66} (GPa)	0.50	21.16	20.36	19.78	3.18	16.78	13.84	16.05
B (GPa)	24.72	11.55	25.29	9.43	20.54	7.30	16.60	6.11
E (GPa)	5.71	27.77	30.42	25.49	4.32	12.76	23.32	19.35
G (GPa)	1.95	12.63	11.70	12.14	1.47	5.28	9.21	9.95
σ	0.46	0.09	0.29	0.04	0.46	0.20	0.26	-0.03
B/G	12.67	0.91	2.16	0.77	13.97	1.38	1.80	0.61

TABLE I. Table S1: Elastic Constants (C_{11}, C_{12}, and C_{66} in GPa), Bulk modulus (B in GPa), Young's modulus (E in GPa), Shear modulus (G in GPa), Poisson's ratio (σ), and Pugh's ratio (B/G) of NbX_2–HfY_2 (X=Se, Te; Y=S, Se) MS vdWHs at strains of -10%, -4%, and +10%.

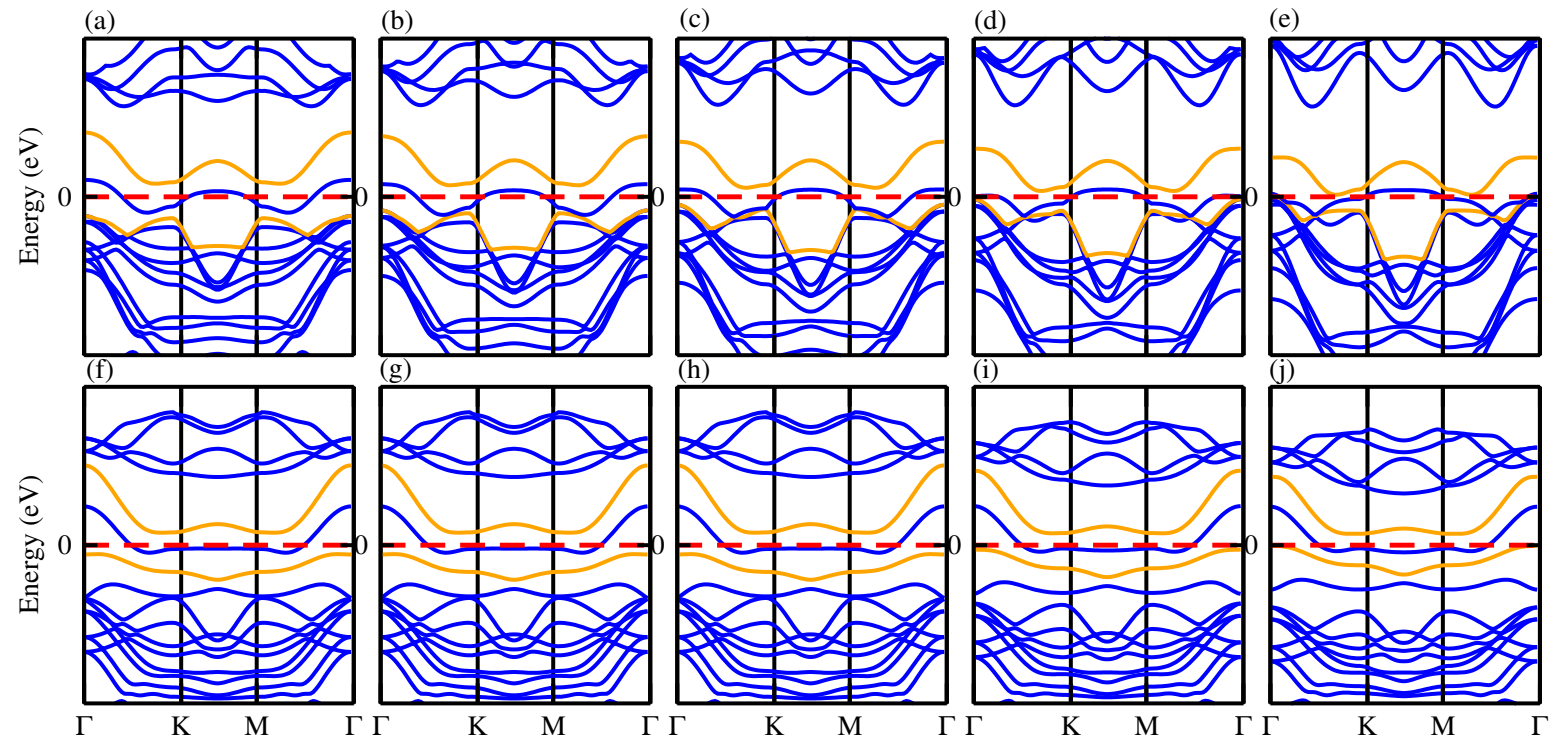

Figure S1: Electronic band structure of $NbSe_2$-HfS_2 under compressive strain (a) -2% (b) -4% (c) -6% (d) -8% (e) -10% and under tensile of (f) 2% (g) 4% (h) 6% (i) 8% and (j) 10%. The blue color represent the electronic states of the metallic $NbSe_2$ layer, while the orange color represent the electronic states of the semiconducting HfS_2 layer.

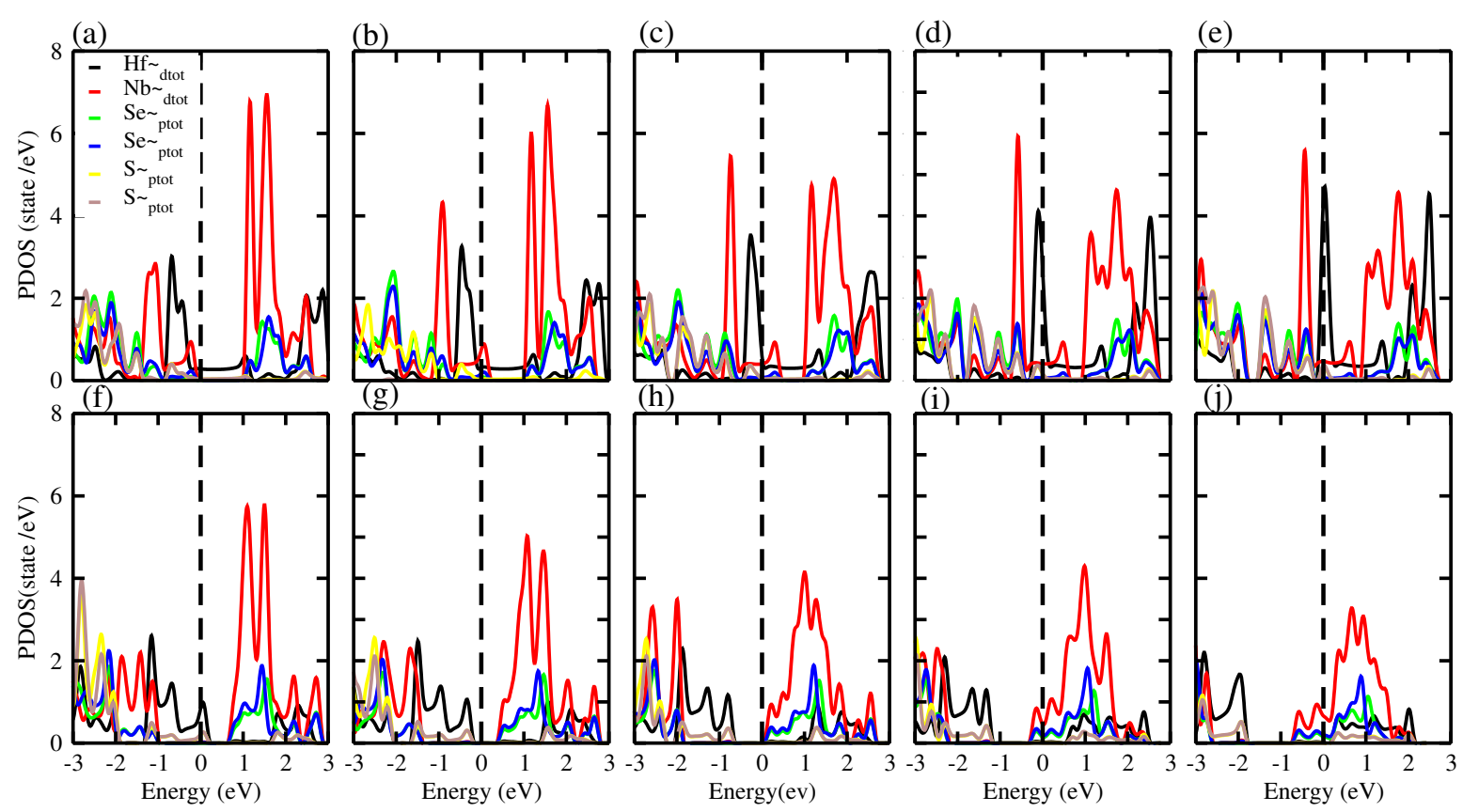

Figure S2: Partial Density of States of $NbSe_2$-HfS_2 under compressive (a) 2% (b) 4% (c) 6% (d) 8% (e) 10% and tensile compression of (f) -2% (g) -4% (h) -6% (i) -8% and (j) -10%.

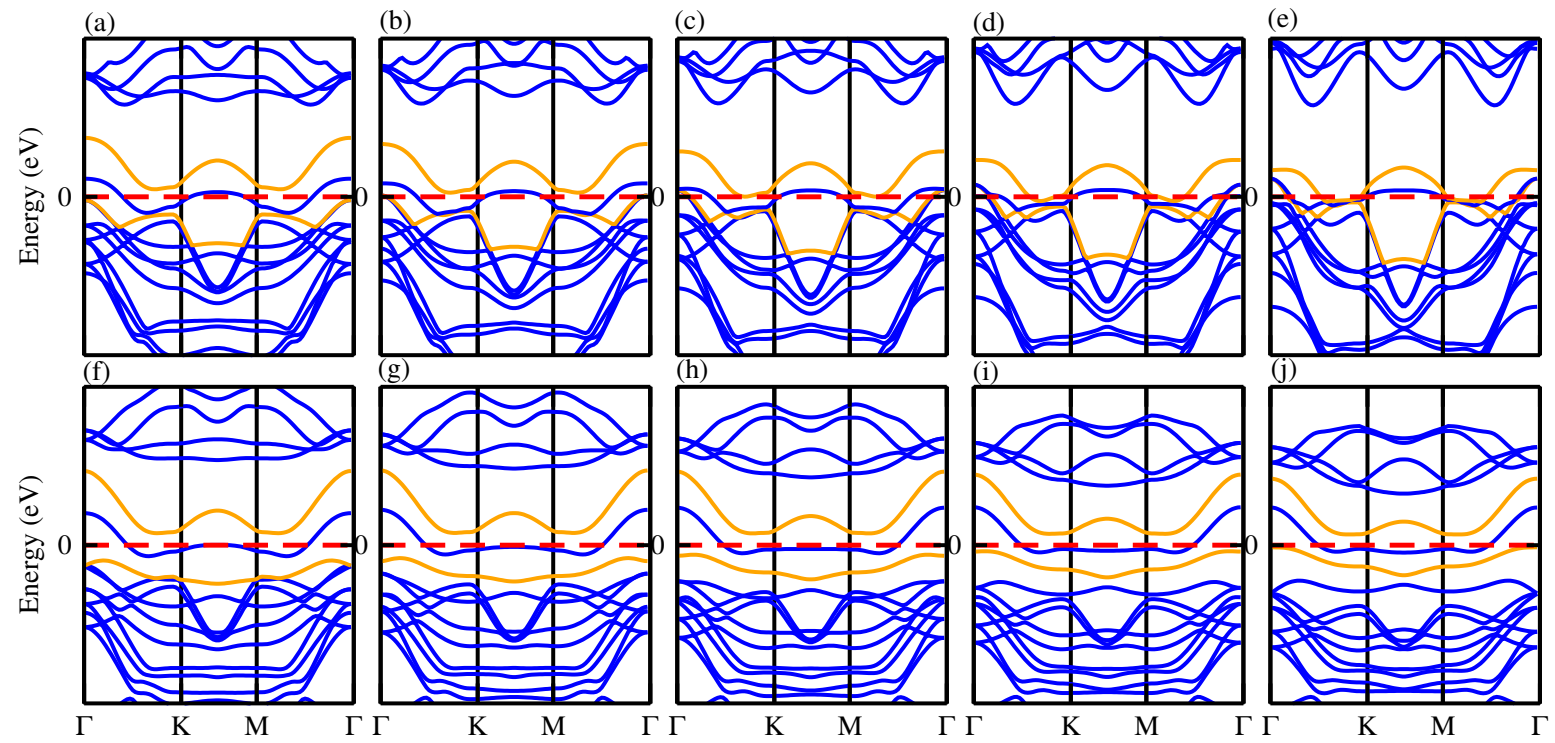

Figure S3: Electronic band structure of $NbSe_2$-$HfSe_2$ under compressive strain (a) -2% (b) -4% (c) -6% (d) -8% (e) -10% and under tensile of (f) 2% (g) 4% (h) 6% (i) 8% and (j) 10%. The blue color represent the electronic states of the metallic $NbSe_2$ layer, while the orange color represent the electronic states of the semiconducting $HfSe_2$ layer.

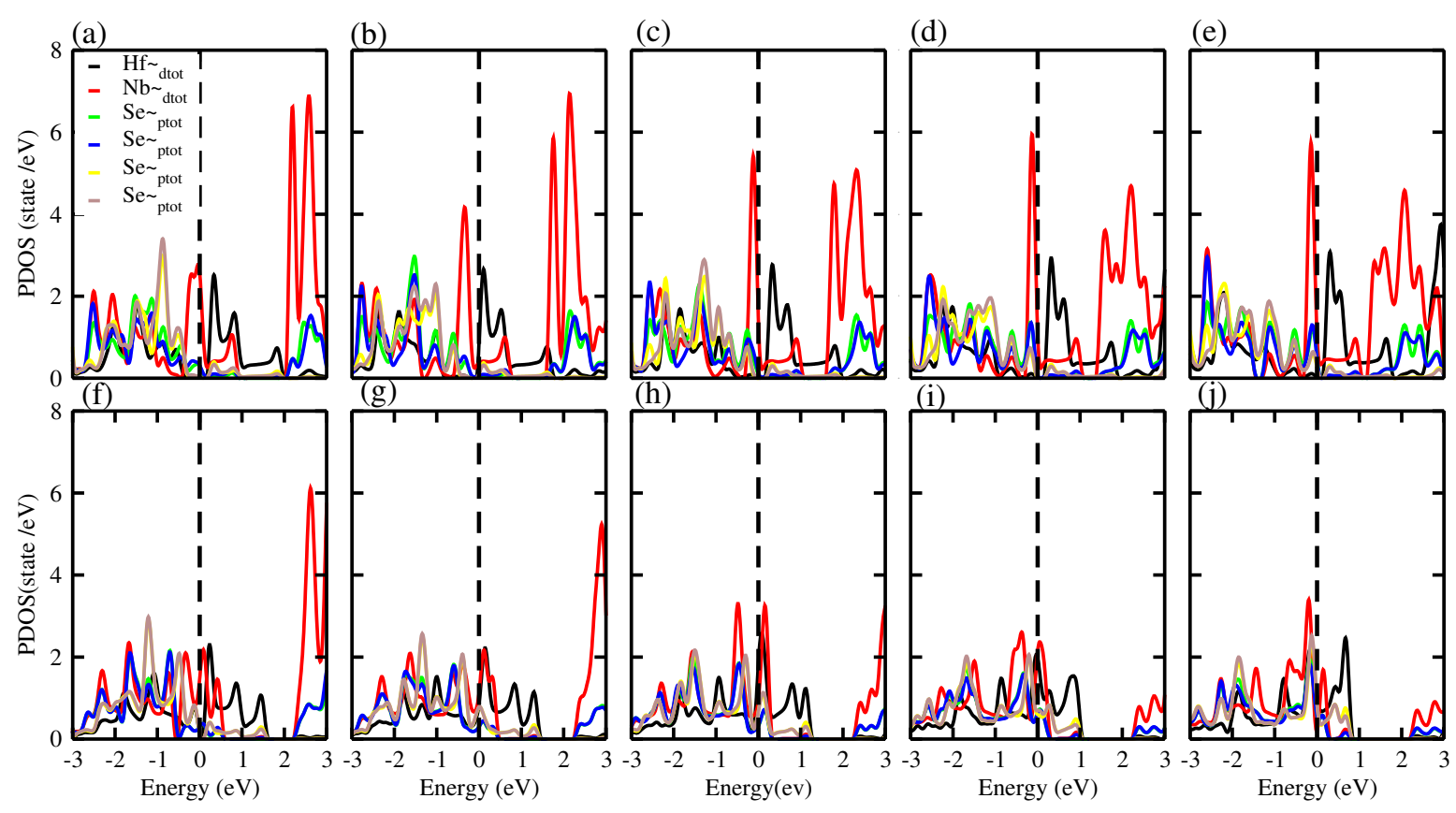

Figure S4: Partial Density of States of $NbSe_2$-$HfSe_2$ under compressive strain (a) -2% (b) -4% (c) -6% (d) -8% (e) -10% and under tensile of (f) 2% (g) 4% (h) 6% (i) 8% and (j) 10%.

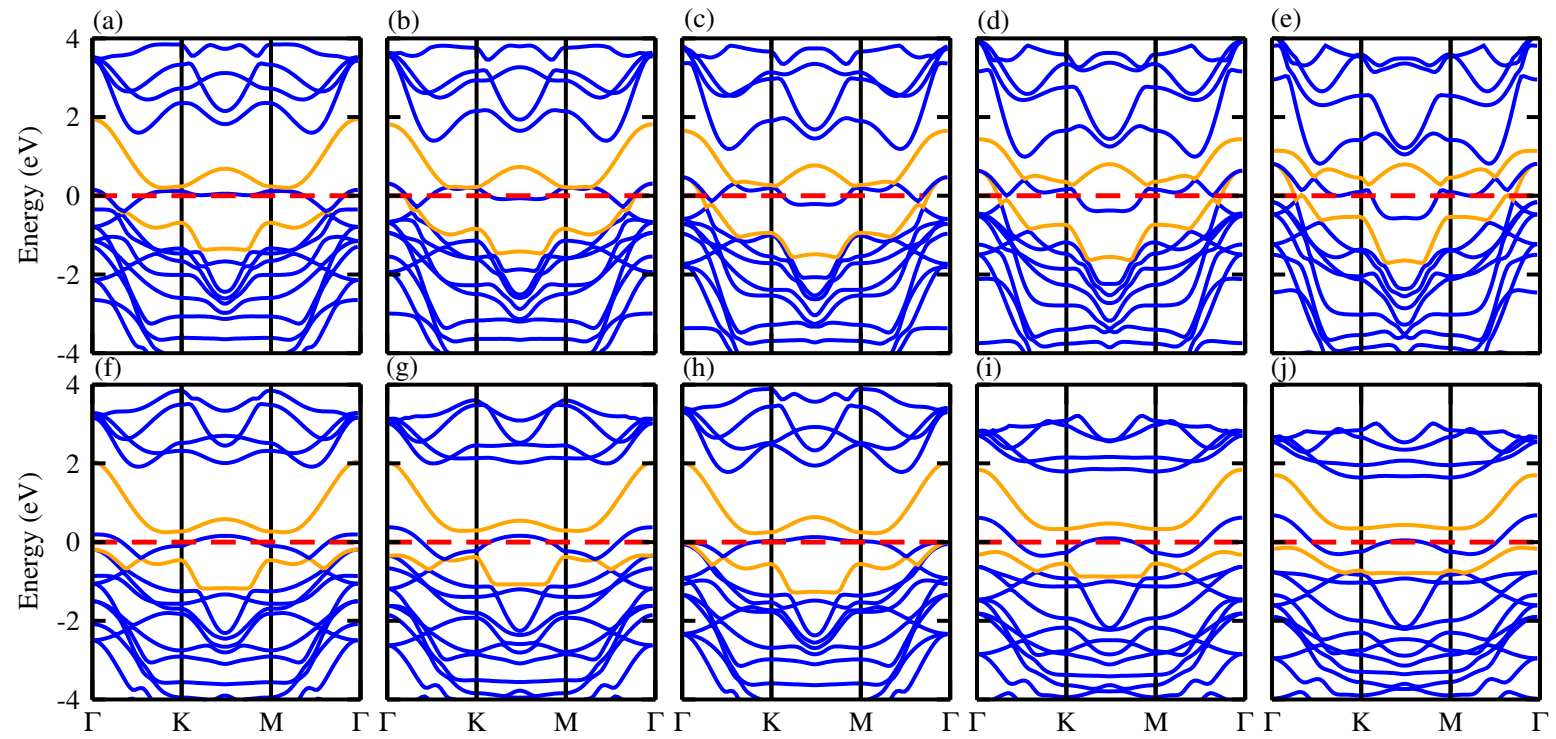

Figure S5: Electronic band structure of $NbTe_2$-HfS_2 under compressive strain (a) -2% (b) -4% (c) -6% (d) -8% (e) -10% and under tensile of (f) 2% (g) 4% (h) 6% (i) 8% and (j) 10%. The blue color represent the electronic states of the metallic $NbTe_2$ layer, while the orange color represent the electronic states of the semiconducting HfS_2 layer.

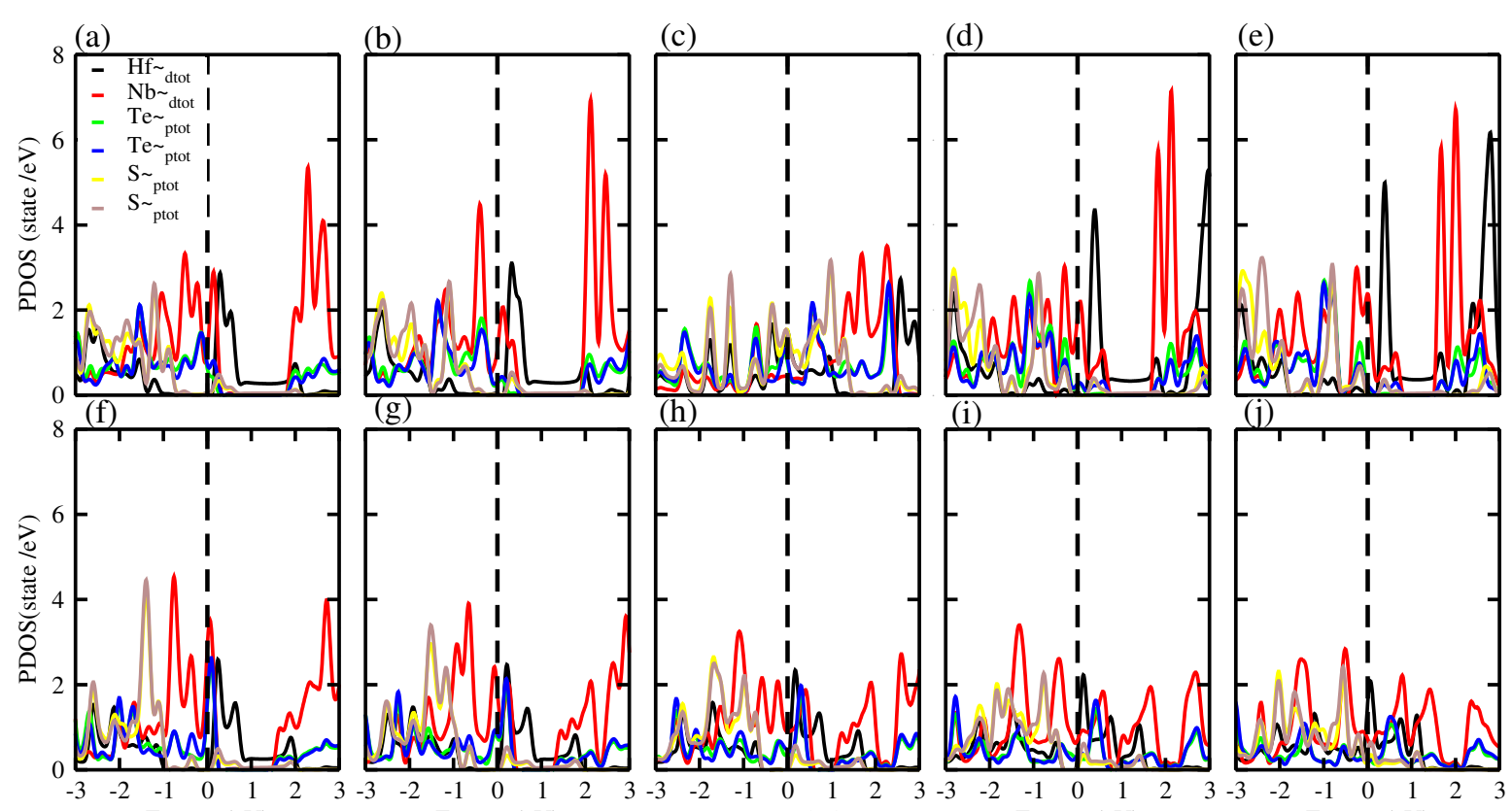

Figure S6: Partial Density of States of $NbTe_2$-HfS_2 under compressive strain (a) -2% (b) -4% (c) -6% (d) -8% (e) -10% and under tensile of (f) 2% (g) 4% (h) 6% (i) 8% and (j) 10%.

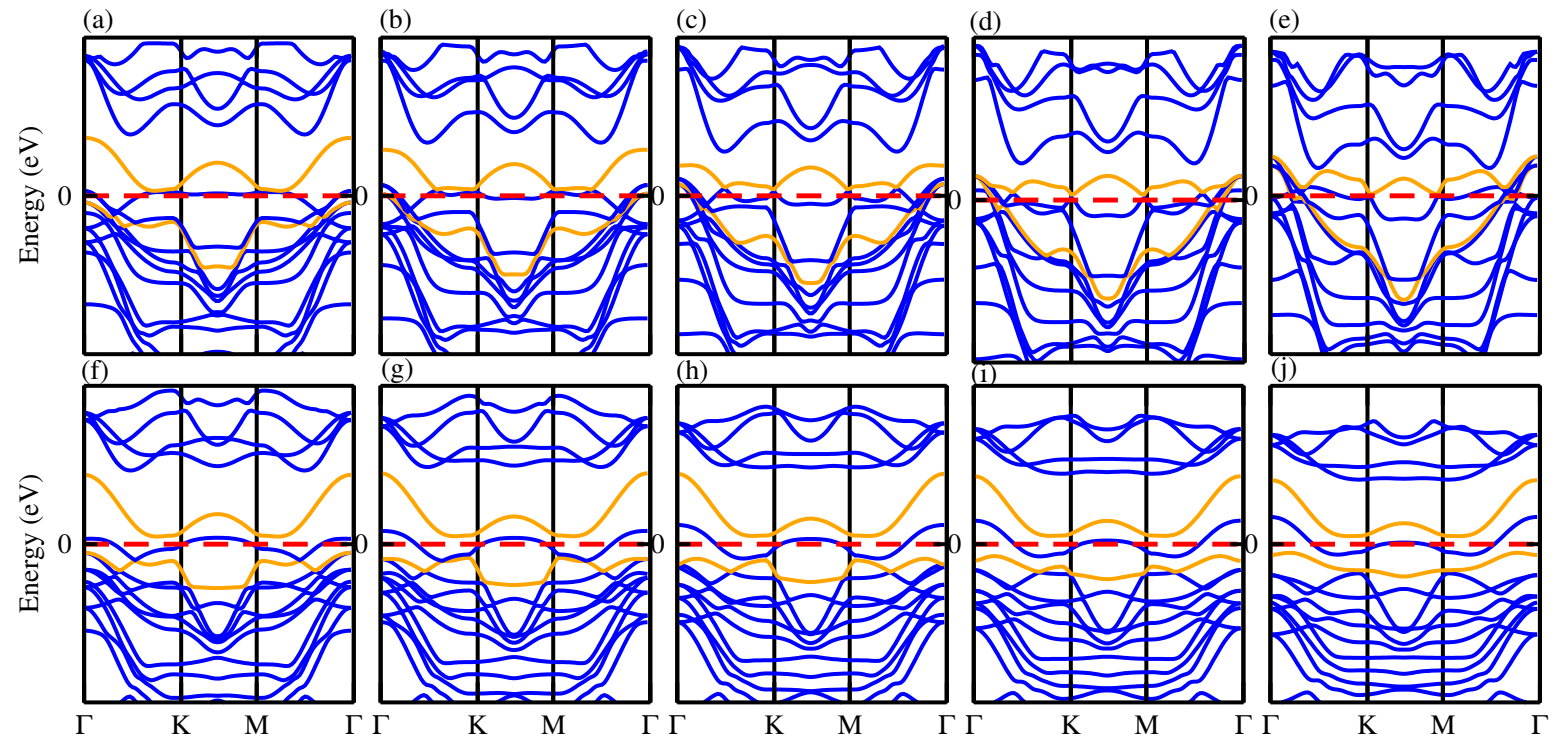

Figure S7: Electronic band structure of $NbTe_2$-$HfSe_2$ under compressive strain (a) -2% (b) -4% (c) -6% (d) -8% (e) -10% and under tensile of (f) 2% (g) 4% (h) 6% (i) 8% and (j) 10% . The blue color represent the electronic states of the metallic $NbTe_2$ layer, while the orange color represent the electronic states of the semiconducting $HfSe_2$ layer.

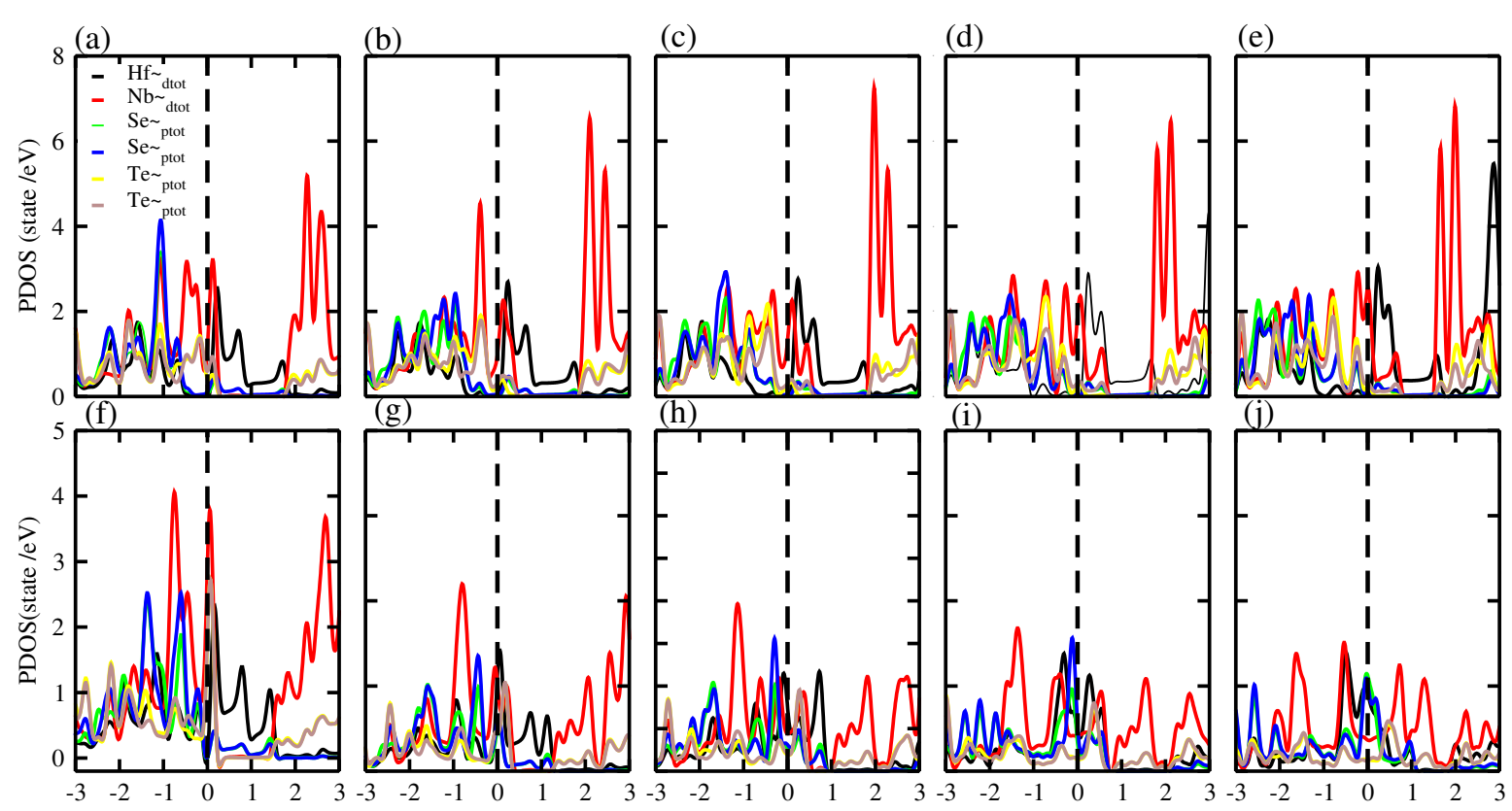

Figure S8: Partial Density of States of $NbTe_2$-$HfSe_2$ under compressive strain (a) -2% (b) -4% (c) -6% (d) -8% (e) -10% and under tensile of (f) 2% (g) 4% (h) 6% (i) 8% and (j) 10%.

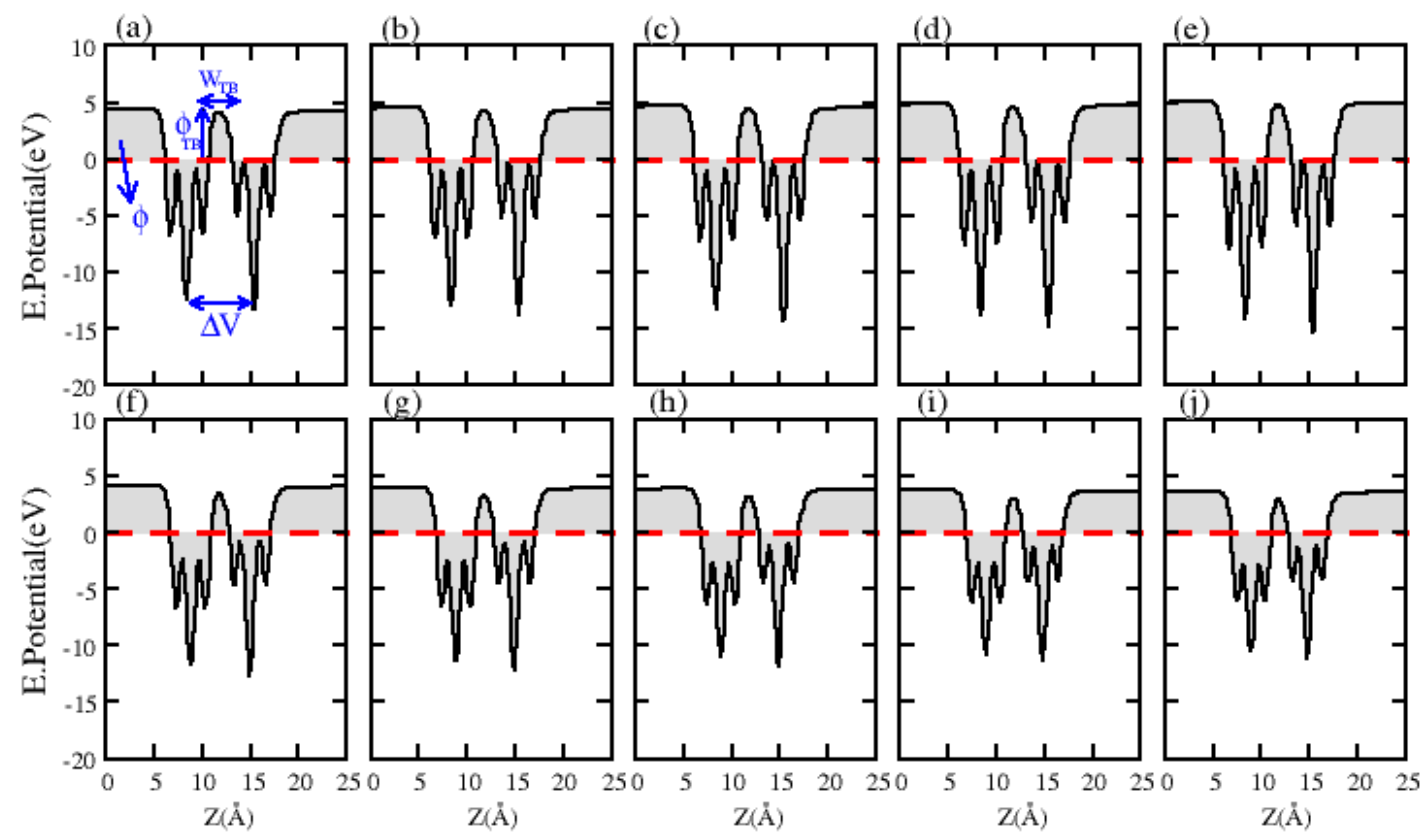

Figure S9: Effective Electrostatic Potential of $NbSe_2$-HfS_2 under compressive strain (a) -2% (b) -4% (c) -6% (d) -8% (e) -10% and under tensile of (f) 2% (g) 4% (h) 6% (i) 8% and (j) 10%.

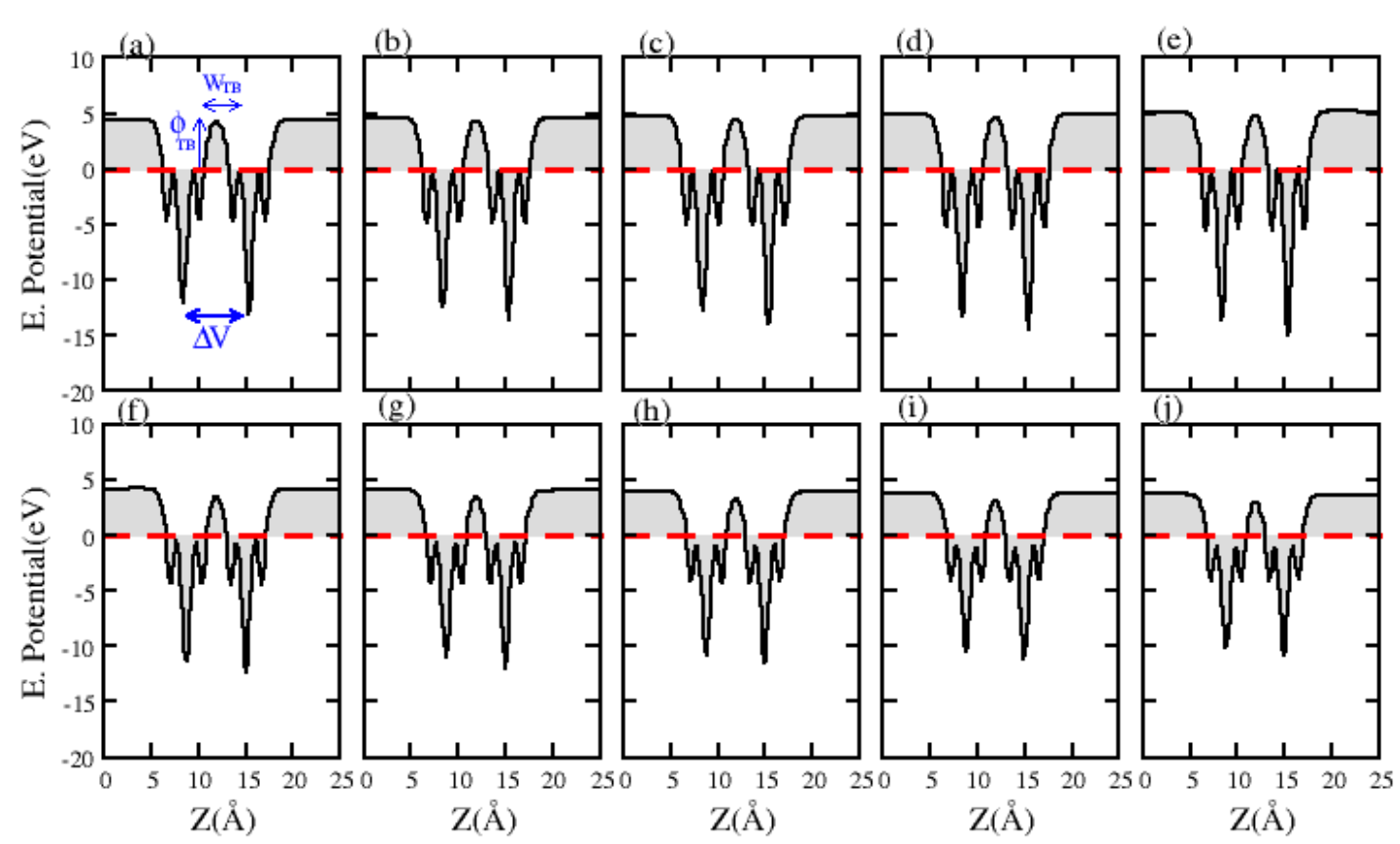

Figure S10: Effective Electrostatic Potential of $NbSe_2$-$HfSe_2$ under compressive strain (a) -2% (b) -4% (c) -6% (d) -8% (e) -10% and under tensile of (f) 2% (g) 4% (h) 6% (i) 8% and (j) 10%.

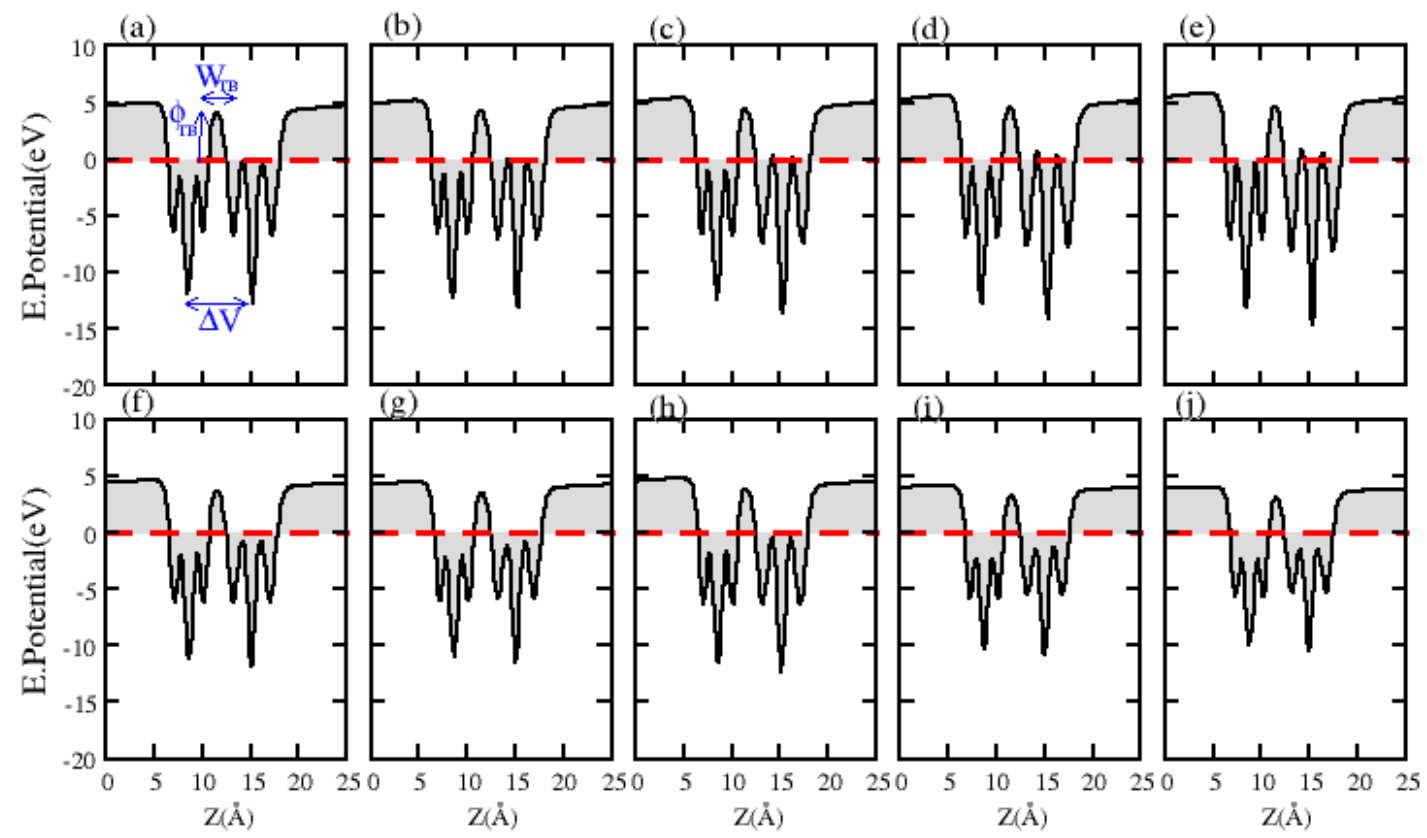

Figure S11: Effective Electrostatic Potential of $NbTe_2$-HfS_2 under compressive strain (a) -2% (b) -4% (c) -6% (d) -8% (e) -10% and under tensile of (f) 2% (g) 4% (h) 6% (i) 8% and (j) 10%.

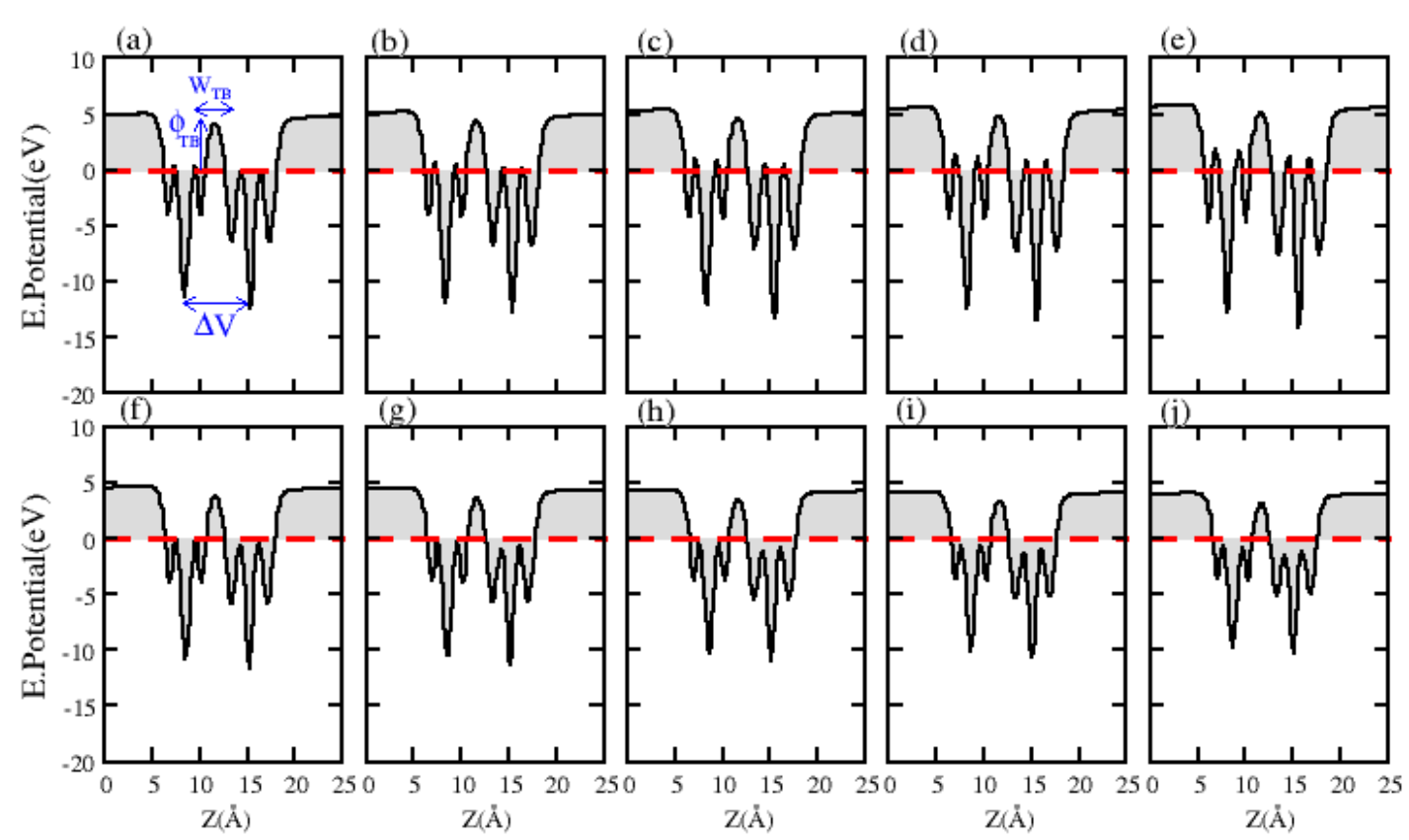

Figure S12: Effective Electrostatic Potential of $NbTe_2$-$HfSe_2$ under compressive strain (a) -2% (b) -4% (c) -6% (d) -8% (e) -10% and under tensile of (f) 2% (g) 4% (h) 6% (i) 8% and (j) 10%.

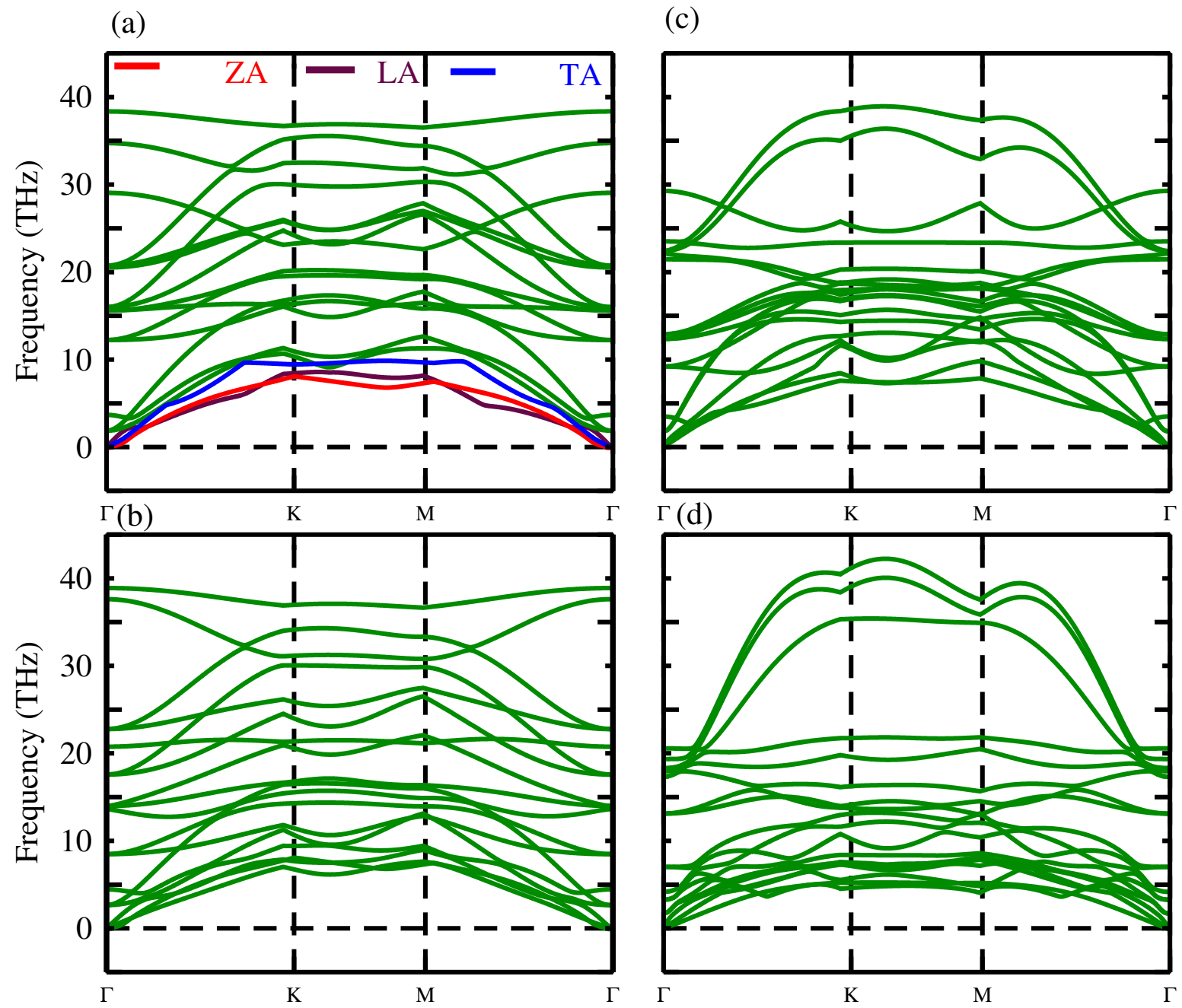

Figure S13: Phonon dispersion of $NbSe_2$–$HfSe_2$ at (a) -4%, (b) 10% and of $NbTe_2$–$HfSe_2$ at (c) -4%, (d)10%.

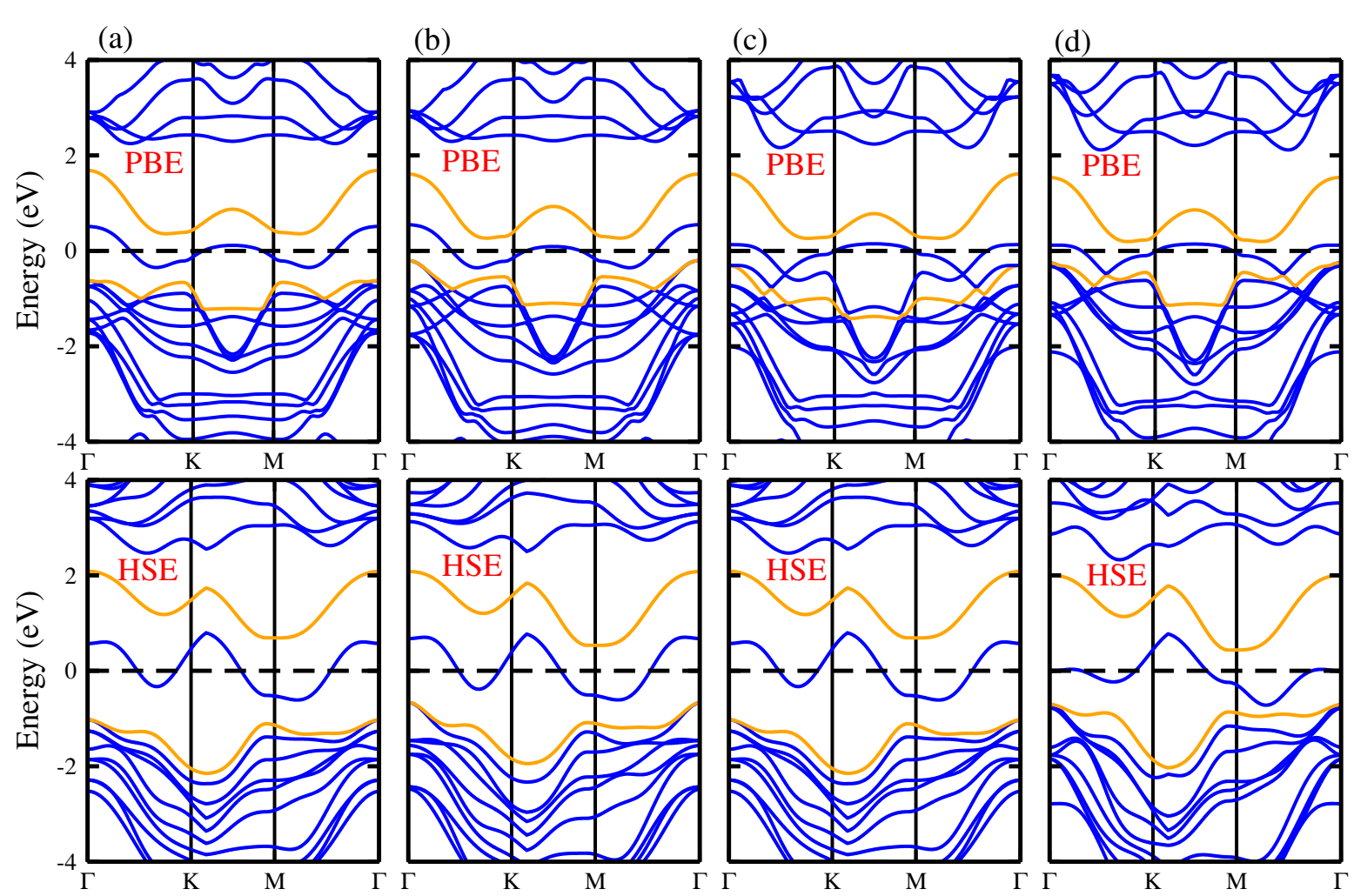

Figure S14: Comparison of PBE and HSE06 calculations of (a) $NbSe_2$–HfS_2, (b) $NbSe_2$-$HfSe_2$, (c) $NbTe_2$-HfS_2 and (d) $NbTe_2$-$HfSe_2$

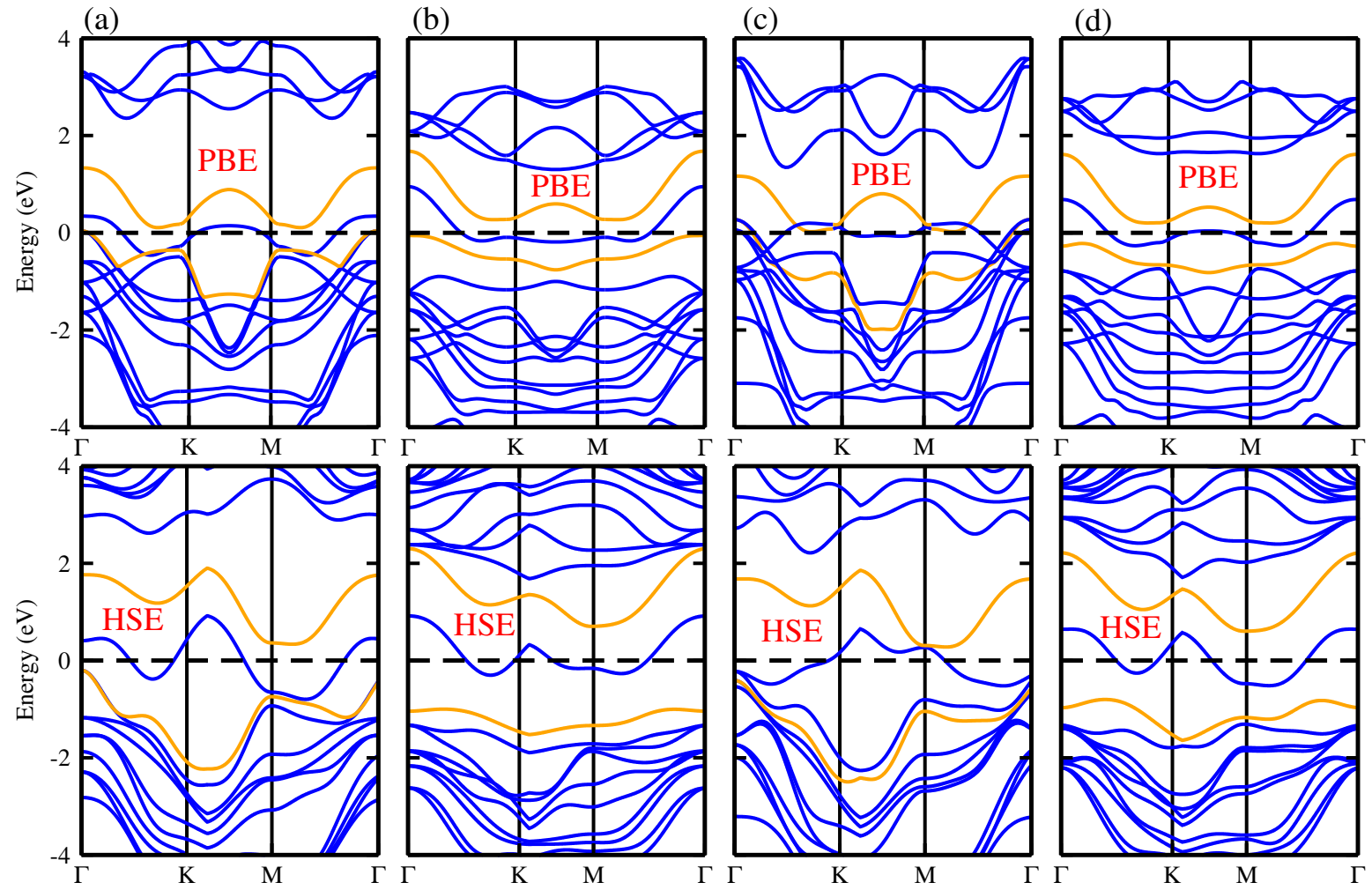

Figure S15: Comparison of PBE and HSE06 calculations of $NbSe_2$-$HfSe_2$ at (a) -4%, and (b) at 10% and of $NbTe_2$–$HfSe_2$ at (c) -4% and (d) at 10%
